# The Plant Defense Signal Salicylic Acid Activates the RpfB-Dependent Quorum Sensing Signal Turnover via Altering the Culture and Cytoplasmic pH in the Phytopathogen Xanthomonas campestris

**DOI:** 10.1128/mbio.03644-21

**Published:** 2022-03-07

**Authors:** Kai Song, Bo Chen, Ying Cui, Lian Zhou, Kok-Gan Chan, Hong-Yan Zhang, Ya-Wen He

**Affiliations:** a State Key Laboratory of Microbial Metabolism, Joint International Research Laboratory of Metabolic and Developmental Sciences, SJTU-NLBP Joint R&D Center on Biopesticides and Biofertilizers, School of Life Sciences and Biotechnology, Shanghai Jiao Tong University (SJTU), Shanghai, China; b Zhiyuan Innovative Research Center, Shanghai Jiao Tong University, Shanghai, China; c Institute of Biological Sciences, Faculty of Science, University of Malaya, Kuala Lumpur, Malaysia; d Faculty of Applied Sciences, UCSI University, Kuala Lumpur, Malaysia; e Shanghai Nong Le Biological Products Company Limited (NLBP), Shanghai, China; University of Nebraska-Lincoln

**Keywords:** DSF, host-pathogen interaction, quorum-sensing signal turnover, RpfB, salicylic acid, *Xanthomonas campestris*

## Abstract

Plant colonization by phytopathogens is a very complex process in which numerous factors are involved. Upon infection by phytopathogens, plants produce salicylic acid (SA) that triggers gene expression within the plant to counter the invading pathogens. The present study demonstrated that SA signal also directly acts on the quorum-sensing (QS) system of the invading pathogen Xanthomonas campestris pv. *campestris* to affect its virulence by inducing turnover of the diffusible signaling factor (DSF) family QS signal. First, Xanthomonas campestris pv. *campestris* infection induces SA biosynthesis in the cabbage host plant. SA cannot be degraded by Xanthomonas campestris pv. *campestris* during culturing. Exogenous addition of SA or endogenous production of SA induces DSF signal turnover during late growth phase of Xanthomonas campestris pv. *campestris* in XYS medium that mimics plant vascular environments. Further, the DSF turnover gene *rpfB* is required for SA induction of DSF turnover. However, SA does not affect the expression of *rpfB* and DSF biosynthesis gene *rpfF* at the transcriptional level. SA induction of DSF turnover only occurs under acidic conditions in XYS medium. Furthermore, addition of SA to XYS medium significantly increased both culture and cytoplasmic pH. Increased cytoplasmic pH induced DSF turnover in a *rpfB*-dependent manner. *In vitro* RpfB-dependent DSF turnover activity increased when pH increased from 6 to 8. SA exposure did not affect the RpfB-dependent DSF turnover *in vitro*. Finally, SA-treated Xanthomonas campestris pv. *campestris* strain exhibited enhanced virulence when inoculated on cabbage. These results provide new insight into the roles of SA in host plants and the molecular interactions between Xanthomonas campestris pv. *campestris* and cruciferous plants.

## INTRODUCTION

Plants are sessile organisms that are prone to infection by diverse pathogens and pests. To survive infections, plants have evolved robust and effective defense mechanisms (1–3). Salicylic acid (SA) is a phenolic acid plant hormone that plays an essential role in plant defenses against biotrophic and semibiotrophic pathogens (4). Substantial progress has been made in understanding the pivotal role of SA in plant immunity over the past 3 decades. Indeed, the biosynthesis, homeostasis, sensing, and SA signaling processes within plants have all been extensively studied (5).

Accumulating evidence also suggests that SA can directly influence the gene expression and metabolic profiles of invading phytopathogens within host-pathogen interactions. For example, exogenous addition of SA decreases expression of the *vir* regulon and activates quormone quenching genes in the model phytopathogen Agrobacterium tumefaciens (6–8). SA also interferes with the transcription of the *repABC* operon and genes associated with quorum sensing (QS) in A. tumefaciens, thereby playing a role in attenuating crown gall disease (9). Moreover, SA can inhibit biofilm formation, motility, and *N*-acyl homoserine lactone-dependent QS machinery in the phytopathogens Pectobacterium carotovorum and Pseudomonas syringae pv. *syringae* (10–11). Further, *hrpA* expression, which encodes a type III pilus, can be severely inhibited by SA *in vitro* in Erwinia amylovora (12). In addition, sodium salicylate has been shown to suppress Xanthomonas oryzae XKK12 swimming in a dose-dependent manner while also inducing EPS production (13). Despite these studies, the roles of SA on the QS-dependent virulence of invading plant pathogens and the associated underlying molecular mechanisms are not yet fully understood.

Xanthomonas campestris pv. *campestris* is the causal agent of black rot, which is a globally important cruciferous plant disease (14). Xanthomonas campestris pv. *campestris* is a vascular pathogen and gains entry into plants via leaf margin hydathodes, stomata, or wounds (15). Once inside the plants, Xanthomonas campestris pv. *campestris* disperses and colonizes the host vascular system via a diffusible signaling factor (DSF)-dependent QS mechanism that regulates a range of virulence factors (14, 16, 17). DSF signaling, biosynthesis, and turnover have all been well studied in Xanthomonas campestris pv. *campestris* (18–20). The DSF, also known as *cis*-11-methyl-dodecenoic acid, and the Burkholderia cenocepacia DSF (BDSF, or *cis-*2-dodecenoic acid) are prominent signals produced by *Xanthomonas* and are synthesized via the classic fatty acid elongation cycle, with RpfF being a key enzyme in biosynthesis (21). A two-component regulatory system, RpfC/RpfG, is involved in DSF signal sensing and transduction (22–24). The activated RpfG features phosphodiesterase activity and degrades the second messenger cyclic di-GMP. Cyclic di-GMP is an inhibitory ligand of the global transcriptional regulator Clp (25). Consequently, the derepressed Clp then further regulates the expression of several hundreds of genes via a hierarchical regulatory network (25, 26). During the late growth stage of Xanthomonas campestris pv. *campestris*, DSF family signal levels sharply decline, leading to *Xanthomonas* exiting the QS stage and shutting down the expression of QS-related genes. This process is achieved by a naturally occurring RpfB-dependent DSF family signal turnover system in Xanthomonas campestris pv. *campestris*, wherein RpfB exhibits fatty acyl-CoA ligase activity and can convert DSF family signals into DSF-CoA via a β-oxidation pathway (27–29). In addition, the DSF signal produced by *Xanthomonas* can interfere with host plant growth, development, and immunity via interkingdom communication in *Arabidopsis*, Nicotiana benthamiana, and rice (30–32). These results suggest that the DSF-dependent QS system plays a key role in Xanthomonas campestris pv. *campestris* infection of host plants and could be an ideal target to develop novel antivirulence therapies.

In this study, Xanthomonas campestris pv. *campestris* infection of cabbage significantly induced SA production. In turn, SA directly acted on the QS activity of the invading pathogens to induce DSF signal turnover. Further analysis identified an acidic pH- and a xylem fluid-like medium-mediated signaling pathway that activates RpfB-dependent DSF turnover. Finally, the SA-treated Xanthomonas campestris pv. *campestris* strain exhibited increased virulence within cabbage. These results provide new insights into the roles of SA in plant-pathogen interactions and the molecular interactions between host plants and the bacterial pathogen Xanthomonas campestris pv. *campestris*.

## RESULTS

### Xanthomonas campestris pv. *campestris* infection promotes SA biosynthesis in cabbage.

**FIG 1:**
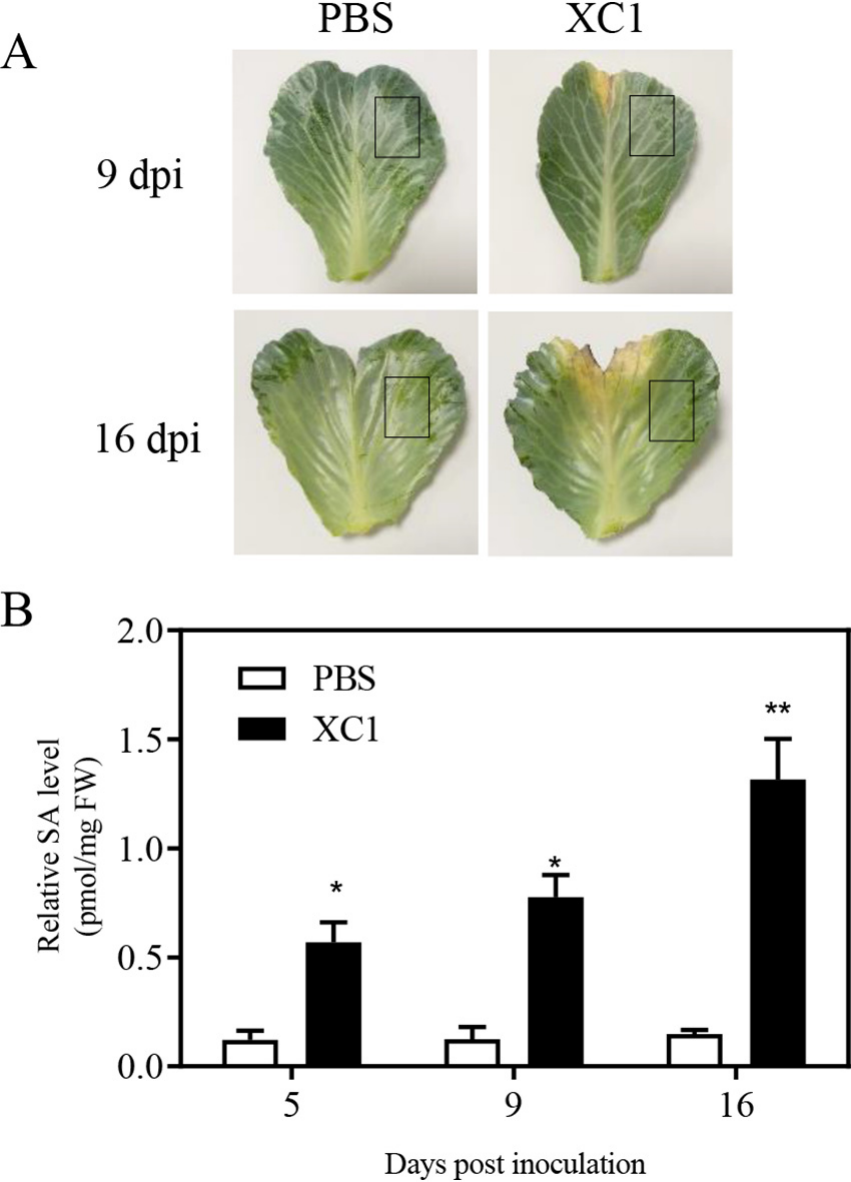
Xanthomonas campestris pv. *campestris* strain XC1 infection in cabbage stimulates SA biosynthesis. (A) Infection lesion development after XC1 inoculation in mature leaves of Brassica oleracea L. (Jingfeng-1) at 9 and 16 days postinoculation. Squares indicate the leaf area for SA analysis. (B) Relative SA levels in cabbage leaf tissues at 5, 9, and 16 days postinoculation with XC1 or phosphate-buffered saline (PBS; 1×, pH 7.4), expressed as molecular concentration per fresh weight (FW). Two independent experiments were conducted, and averages of 5 leaves, along with standard deviations obtained, are shown. Statistically significant differences are indicated (*, *P ≤ *0.05; **, *P ≤ *0.01).

Two-month mature cabbages were infected by wild-type XC1 bacteria using the scissor-clipping inoculation method. Clear infection lesions were observed 9 days postinoculation (dpi) in cabbage leaves (Fig. 1A). No infection lesions were observed in control phosphate-buffered saline (PBS)-inoculated leaves (Fig. 1A). High-performance liquid chromatography coupled with triple-quadrupole tandem mass spectrometry (HPLC-QqQ-MS/MS) was used to quantitatively assess endogenous SA levels in cabbage leaf tissues (Fig. S1A to C in the supplemental material). SA levels in XC1-infected cabbage leaves were 0.59 pmol/mg fresh weight (FW) at 5 dpi, 0.74 pmol/mg FW at 9 dpi, and 1.31 pmol/mg FW at 16 dpi. These values represent approximately 4.6- to 9.3-fold higher SA levels than observed in the PBS-inoculated leaf tissues at 5, 9, and 16 dpi (Fig. 1B).

10.1128/mbio.03644-21.1 FIG S1 High-performance liquid chromatography coupled with triple-quadrupole tandem mass spectrometry (HPLC-QqQ-MS/MS) and HPLC-based quantitative analysis of SA level. (A) MS spectra of SA at 0.1 μM to 1.0 μM dissolved in ethanol. (B) The plot between the peak area (A) and SA concentration (S_A_). (C) HPLC-QqQ-MS/MS spectrum of SA in the crude cabbage extract. (D) HPLC spectra of SA at 0.1 mM to 10.0 mM dissolved in ethanol. (E) The plot between the peak area (A) of the chromatogram and SA concentration (S_A_). (F) HPLC analysis of SA in the crude extract from Xanthomonas campestris pv. *campestris* culture. (G) Relative SA levels in cabbage leaf tissues at 5, 9, and 16 days postinoculation with 1× PBS and E. coli DH5α strain, expressed as molecular concentration per fresh weight (FW). Two independent experiments were conducted, and averages of 5 leaves, along with standard deviations obtained, are shown. Download FIG S1, TIF file, 1.2 MB. Copyright © 2022 Song et al. 2022 Song et al. https://creativecommons.org/licenses/by/4.0/ This content is distributed under the terms of the Creative Commons Attribution 4.0 International license.

### Exogenous addition of SA induces DSF and BDSF turnover.

Xanthomonas campestris pv. *campestris* is a vascular pathogen. Thus, to understand how Xanthomonas campestris pv. *campestris* survives SA stress during infection inside the host plant, XYS, a specially designed medium that mimics the within-plant growth conditions experienced by Xanthomonas campestris pv. *campestris* during infection (21), was used for Xanthomonas campestris pv. *campestris* growth. Our previous results showed that Xanthomonas campestris pv. *campestris* could utilize and degrade SA analogues, 3-hydroxybenzoic acid (3-HBA) and 4-HBA (33, 34), we first sought to determine whether Xanthomonas campestris pv. *campestris* can degrade SA *in vitro*. To this aim, an HPLC-based method for quantitative analysis of SA level in cultures of different Xanthomonas campestris pv. *campestris* strains was established in our study (Fig. S1D and E). SA was added into medium XYS at a final concentration of 100 μM, and SA levels in the cultures of XC1, DSF-overproducing Δ*rpfC* mutant, and DSF/BDSF turnover mutant Δ*rpfB* remained relatively stable (Fig. S2), suggesting that SA was not degraded by either of these strains.

10.1128/mbio.03644-21.2 FIG S2 SA is not degraded in XYS cultures of XC1, Δ*rpfC*, and Δ*rpfB*. (A) The growth-time curve of Xanthomonas campestris pv. *campestris* strains in XYS medium supplemented with 100 μM SA. (B) SA levels in XYS cultures. Shown are the averages for three technical repeats with standard deviations. Download FIG S2, TIF file, 0.4 MB. Copyright © 2022 Song et al. 2022 Song et al. https://creativecommons.org/licenses/by/4.0/ This content is distributed under the terms of the Creative Commons Attribution 4.0 International license.

**FIG 2:**
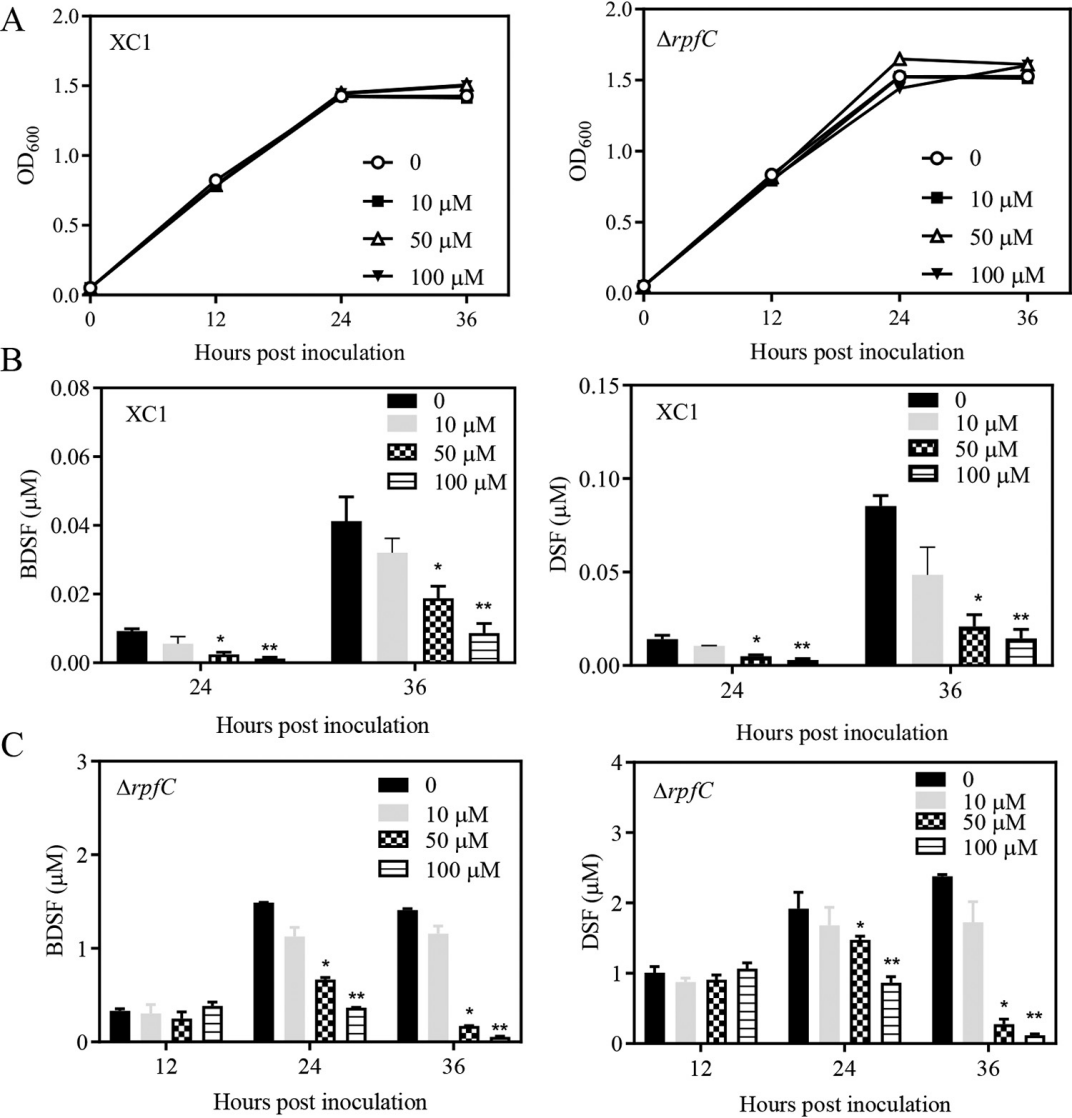
Exogenous addition of SA induces BDSF and DSF turnover in the strains XC1 and Δ*rpfC*. (A) Growth time course of wild-type XC1 or Δ*rpfC* in XYS medium supplemented with 10 to 100 μM SA. (B) BDSF and DSF levels in XC1 XYS cultures supplemented with 10 to 100 μM SA. (C) BDSF and DSF levels in Δ*rpfC* XYS cultures supplemented with 10 to 100 μM SA. Three independent experiments were conducted, and averages, along with standard deviations, are shown. Statistically significant differences are indicated (*, *P ≤ *0.05; **, *P ≤ *0.01).

Next, we assessed how Xanthomonas campestris pv. *campestris* would respond to SA. SA was added to XYS medium at final concentrations of 10 μM, 50 μM, and 100 μM, respectively. Exogenous addition of 10 to 100 μM SA had little effect on XC1 growth (Fig. 2A). Addition of 10 μM SA to XYS culture of XC1 had no significant effect on BDSF and DSF turnover (Fig. 2B). However, addition of 50 to 100 μM SA to XYS cultures of XC1 significantly induced BDSF and DSF turnover at 24 and 36 h postinoculation (hpi) in a dosage-dependent manner (Fig. 2B). For example, the DSF levels at 36 hpi were 0.033, 0.020, and 0.014 μM in the presence of 10, 50 μM, and 100 μM SA, respectively, representing about 38.8%, 23.5%, and 16.4%, respectively, of the basal concentration (0.085 μM) observed in the absence of SA (Fig. 2B). DSF and BDSF levels at 12 hpi in the wild-type strain XC1 were extremely low and undetectable (data not shown).

Further investigation was conducted to test whether exogenous application of SA induces BDSF and DSF turnover in the DSF-overproducing strain Δ*rpfC*. Addition of 10 μM SA to XYS medium did not significantly affect BDSF or DSF levels of Δ*rpfC* (Fig. 2C). Moreover, exogenous addition of 50 to 100 μM SA did not significantly affect DSF and BDSF levels of Δ*rpfC* in XYS medium at 12 hpi but significantly decreased DSF and BDSF levels at 24 and 36 hpi in a concentration-dependent manner (Fig. 2C). The BDSF levels in Δ*rpfC* cultures supplemented with 50 or 100 μM SA at 36 hpi were 0.17 μM and 0.05 μM, respectively. These concentrations represented 12.1% and 3.5% of the basal concentration (1.40 μM) in Δ*rpfC* cultures in the absence of SA (Fig. 2C).

### Endogenous production of SA induces DSF and BDSF turnover.

**FIG 3:**
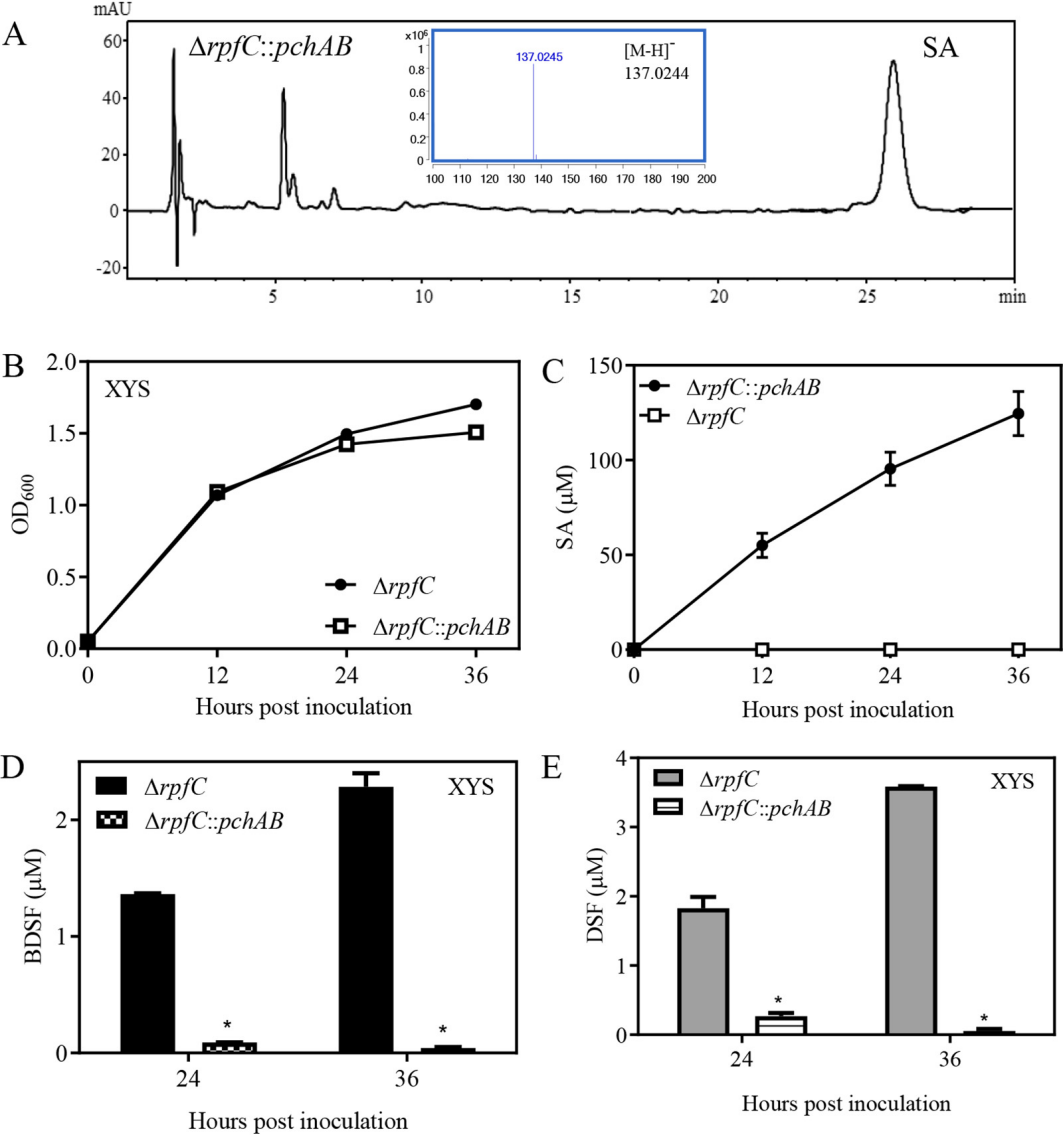
Endogenous production of SA induces BDSF and DSF turnover. (A) Verification of SA production in strain Δ*rpfC*::*pchAB* by HPLC and mass spectrometry assay. (B) Δ*rpfC* and Δ*rpfC*::*pchAB* growth over time in XYS medium. (C) SA levels in Δ*rpfC* and Δ*rpfC*::*pchAB* cultures in XYS medium. (D and E) BDSF and DSF levels in Δ*rpfC* and Δ*rpfC*::*pchAB* cultures in XYS medium 24 h and 36 h after inoculation. Three independent experiments were conducted, and averages, along with standard deviations, are shown. Statistically significant differences are indicated (*, *P ≤ *0.05).

Our previous results showed that Xanthomonas campestris pv. *campestris* cells have the ability to take up the phenolic acids such as 3-HBA and 4-HBA via an unknown mechanism (35, 36). To further verify the effect of SA on DSF and BDSF turnover in Xanthomonas campestris pv. *campestris*, an SA-producing strain, Δ*rpfC*::*pchAB*, was generated by integrating the *pchAB* gene cluster into the Δ*rpfC* chromosome (Fig. 3A). *pchAB* is responsible for the production of SA, a precursor for pyochelin, in Pseudomonas aeruginosa (37). When the Δ*rpfC* and Δ*rpfC*::*pchAB* strains were grown in XYS medium, SA levels in Δ*rpfC*::*pchAB* cultures were 55.1 μM at 12 hpi, 95.5 μM at 24 hpi, and 124.6 μM at 36 hpi, while SA was not detected in Δ*rpfC* cultures (Fig. 3A to C). There were no significant differences in DSF and BDSF levels at 12 hpi in XYS medium between the two strains. However, DSF and BDSF levels in the Δ*rpfC*::*pchAB* cultures at 24 hpi and 36 hpi were significantly lower than those of the Δ*rpfC* strain (Fig. 3D and E). For example, the DSF level of Δ*rpfC*::*pchAB* at 36 hpi was 0.05 μM, which is only 1.4% of the concentration of 3.58 μM achieved by the Δ*rpfC* strain (Fig. 3E). The BDSF level of Δ*rpfC*::*pchAB* at 36 hpi was 0.04 μM, which is about 1.9% of the 2.28 μM achieved by the Δ*rpfC* strain (Fig. 3D). These results suggest that endogenous SA production induces DSF and BDSF turnover in XYS medium.

### SA-induced DSF and BDSF turnover is dependent on rpfB.

**FIG 4:**
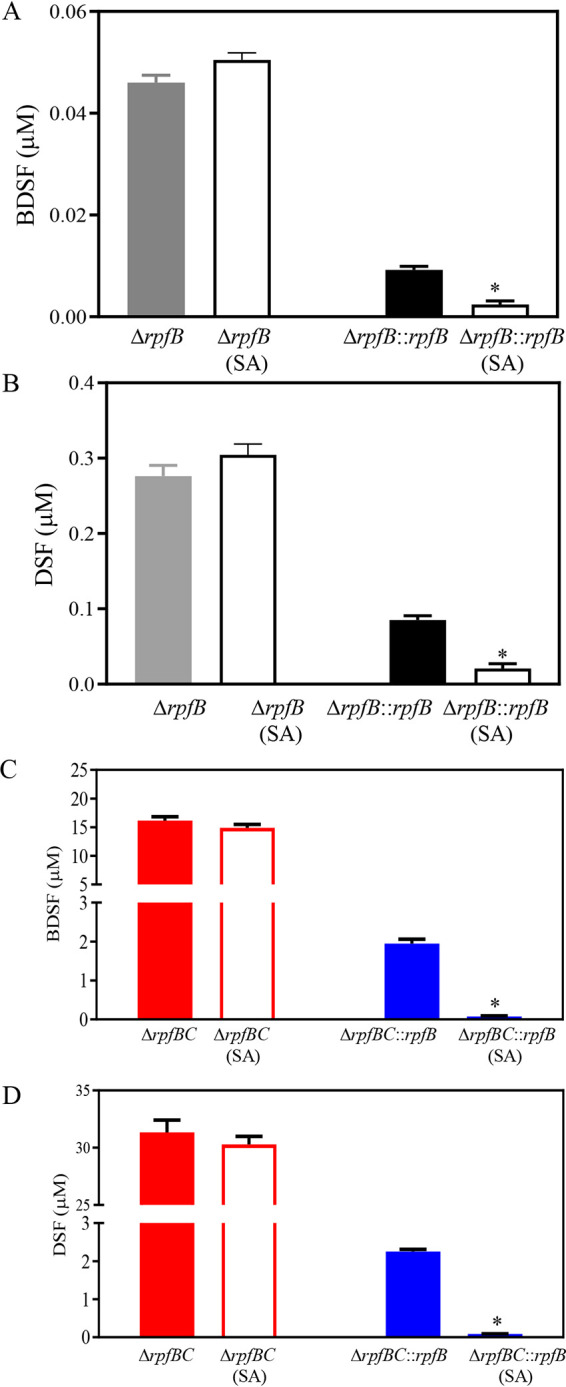
*rpfB* is required for SA-induced DSF and BDSF turnover. (A and B) BDSF and DSF levels in strains Δ*rpfB* and Δ*rpfB*::*rpfB* (Δ*rpfB* strain complemented by a single copy of *rpfB* integrated into the genome) in XYS medium supplemented with 100 μM at 24 hpi. (C and D) BDSF and DSF levels in *rpfC* and *rpfB* double mutant Δ*rpfBC* and Δ*rpfBC*::*rpfB* (Δ*rpfBC* strain complemented by a single copy of *rpfB* integrated into the genome) grown in XYS medium supplemented with 0 or 50 μM SA at 24 hpi. Three independent experiments were conducted, and averages, along with standard deviations, are shown. Statistically significant differences are indicated (*, *P ≤ *0.05).

*rpfB* encodes a long-chain fatty acid CoA ligase and is one of the key enzymes responsible for DSF and BDSF turnover in Xanthomonas campestris pv. *campestris* (28). To investigate whether *rpfB* is required for SA-induced DSF and BDSF turnover, several strains were evaluated, including Δ*rpfB*, Δ*rpfB* complemented with a single copy of *rpfB* at the *att*Tn7 genomic site (Δ*rpfB*::*rpfB* here), an *rpfB* and *rpfC* double deletion mutant (referred to as Δ*rpfBC* here), and Δ*rpfBC* complemented with a single copy of *rpfB* at the *att*Tn7 genomic site (referred to as Δ*rpfBC*::*rpfB* here). BDSF and DSF levels of strains Δ*rpfB* and Δ*rpfB*::*rpfB* were compared in the absence and presence of 100 μM SA in XYS medium. Addition of 100 μM SA failed to induce DSF and BDSF turnover in strain Δ*rpfB* but significantly induced DSF and BDSF turnover in strain Δ*rpfB*::*rpfB* (Fig. 4A and B). For example, the BDSF and DSF levels of Δ*rpfB*::*rpfB* in the presence of SA were 0.002 and 0.02 μM at 36 hpi, respectively, which represented only 22.2% and 25% of these values in the absence of SA (0.009 and 0.08 μM, respectively) (Fig. 4A and B).

Similarly, the BDSF and DSF levels of strains Δ*rpfBC* and Δ*rpfBC*::*rpfB* in XYS medium were also compared in the absence and presence of 100 μM SA. Exogenous addition of 100 μM SA failed to induce BDSF and DSF turnover in Δ*rpfBC* (Fig. 4C and D). In contrast, addition of 100 μM SA significantly induced BDSF and DSF turnover in strain Δ*rpfBC*::*rpfB* (Fig. 4C and D). For example, the DSF and BDSF levels in Δ*rpfBC*::*rpfB* treated with SA were 0.23 and 0.15 μM at 36 hpi, respectively, which are significantly lower than concentrations in the absence of SA (2.2 and 2.1 μM, respectively) (Fig. 4C and D).

### SA does not affect the expression of *rpfB* and *rpfF*.

The levels of DSF family signals in Xanthomonas campestris pv. *campestris* are dependent on the relative activities of RpfF-dependent biosynthesis and RpfB-dependent degradation. To investigate the molecular mechanisms underlying SA-dependent DSF and BDSF turnover, the effects of SA (10 and 100 μM) on the expression of *rpfB* and *rpfF* at the transcriptional level were evaluated. To this end, the reporter strains XC1::P*_rpfB_*-*gusA* and XC1::P*_rpfF_*_-_*gusA* were first generated to monitor *rpfB* and *rpfF* transcriptional activity. No significant differences were observed in *rpfB* transcript levels between XC1::P*_rpfB_*_-_*gusA* and the SA-treated XC1::P*_rpfB_*_-_*gusA* strains (Fig. S3A and B). Similarly, there were no significant differences in *rpfF* transcriptional levels between XC1::P*_rpfF_*_-_*gusA* and SA-treated XC1::P*_rpfF_*_-_*gusA* strains (Fig. S3D and E).

10.1128/mbio.03644-21.3 FIG S3 The *rpfB* and *rpfF* transcriptional and translational levels of Xanthomonas campestris pv. *campestris* strains in XYS medium supplemented with SA. (A) β-Glucuronidase (GUS) activity (MUG as the substrate) of the reporter strain XC1::P*_rpfB_*-*gusA*. (B) GUS activity of the reporter strain XC1::P*_rpfB_*-*gusA* using X-Gluc (5-bromo-4-chloro-3-indolyl-β-d-glucuronic acid) as the substrate. (C) Western blotting to show RpfB protein levels of XC1 and Δ*rpfC*. (D) GUS activity of the reporter strain XC1::P*_rpfF_*-*gusA* using MUG as the substrate. (E) GUS activity of the reporter strain XC1::P*_rpfF_*-*gusA* using X-Gluc as the substrate. (F) Western blotting to show RpfF protein levels of XC1 and Δ*rpfC*. For panels A and D, three independent experiments were conducted, and averages, along with standard deviations, are shown. Download FIG S3, TIF file, 1.7 MB. Copyright © 2022 Song et al. 2022 Song et al. https://creativecommons.org/licenses/by/4.0/ This content is distributed under the terms of the Creative Commons Attribution 4.0 International license.

To investigate the protein levels of RpfB and RpfF in the absence and presence of SA, Western blot analysis was conducted using polyclonal antibodies against RpfB or RpfF (28). No significant differences in RpfB or RpfF protein levels were induced by SA in strains XC1 and Δ*rpfC* (Fig. S3C and F), suggesting that the effect of SA on DSF and BDSF turnover mediated by RpfB did not involve a transcriptional or posttranscriptional regulation of this gene or *rpfF*.

### Xanthomonas campestris pv. *campestris* growth in XYS medium leads to an acidic environment and a lower cytoplasmic pH.

**FIG 5:**
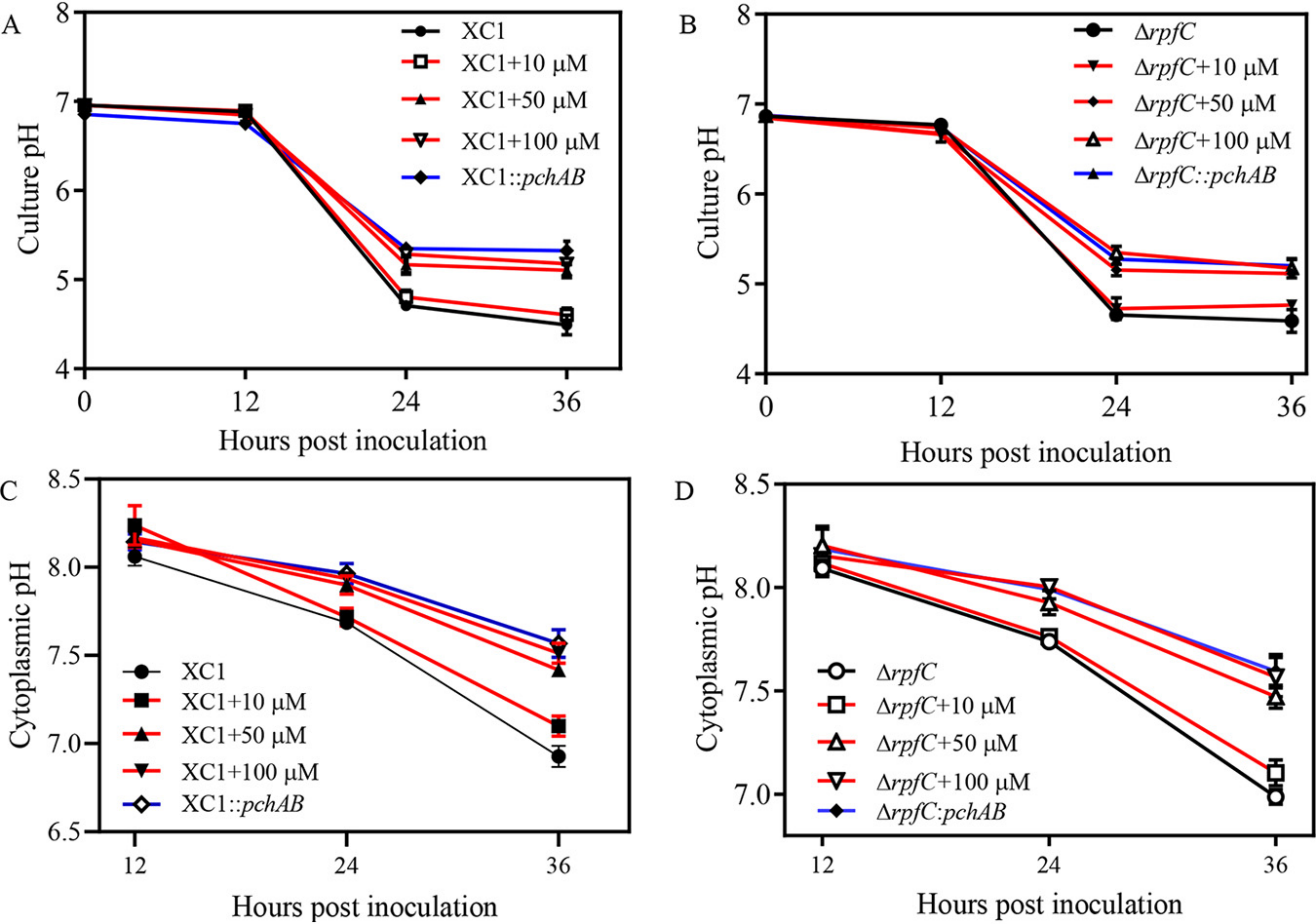
Addition of SA significantly increased the culture and cytoplasmic pH of XC1 or Δ*rpfC*. (A) Culture pH of XC1, XC1 supplemented with 10 to 100 μM SA, and XC1::*pchAB*. (B) Culture pH of Δ*rpfC*, Δ*rpfC* supplemented with 10 to 100 μM SA, and Δ*rpfC*::*pchAB*. (C) Cytoplasmic pH of XC1, XC1 supplemented with 10 to 100 μM SA, and XC1::*pchAB*. (D) Cytoplasmic pH of Δ*rpfC*, Δ*rpfC* supplemented with 10 to 100 μM SA, and Δ*rpfC*::*pchAB.* Three independent experiments were conducted, and averages, along with standard deviations, are shown for three technical replicates.

To further investigate why SA only induces BDSF and DSF turnover in XYS medium, pH dynamic changes in cultures with XC1 and Δ*rpfC* in XYS medium were evaluated by directly measuring culture pH. The culture pH of XC1 in XYS medium decreases from 6.96 at 0 hpi to 6.80 at 12 hpi, 4.71 at 24 hpi, and 4.49 at 36 hpi (Fig. 5A). Culture pH trends for Δ*rpfC* in XYS were similar to those of strain XC1 (Fig. 5B). These results suggest that Xanthomonas campestris pv. *campestris* growth in XYS medium leads to an acidic environment.

The cytoplasmic pH of Xanthomonas campestris pv. *campestris* strains in different media was also investigated using an mCherry-pHluorin translational fusion protein via the recombinant vector pBBR1MCS-2 following methods described by Zarkan et al. (38) (Fig. S4A). pHluorin is a pH-sensitive green fluorescent protein (39), while mCherry fluorescence is not pH sensitive and was therefore used to normalize changes in fluorescence caused by cell density and plasmid copy number (38). The constructed reporter strain pBBR1MCS-2-mCherry-pHluorin was used to establish a standard curve by plotting different cytoplasmic pH and ln(pHluorin/mCherry) in different media (Fig. S4B). The cytoplasmic pH of XC1 and Δ*rpfC* in XYS medium significantly decreased from ∼8.05 to 8.07 at 12 hpi to ∼6.92 to 6.98 at 36 hpi (Fig. 5C and D).

10.1128/mbio.03644-21.4 FIG S4 Reporter plasmid and standard curve for cytoplasmic pH measurement. (A) The recombinant pBBR plasmid expressing mCherry and pHluorin. (B) The standard curve showing the relationship of ln(pHluorin/mCherry) and pH. Download FIG S4, TIF file, 1.1 MB. Copyright © 2022 Song et al. 2022 Song et al. https://creativecommons.org/licenses/by/4.0/ This content is distributed under the terms of the Creative Commons Attribution 4.0 International license.

### Exogenous addition of SA or endogenous production of SA significantly prevents Xanthomonas campestris pv. *campestris* culture pH and cytoplasmic pH decrease.

The culture and cytoplasmic pH of strains XC1 and Δ*rpfC* grown in XYS medium in the presence of 10, 50, and 100 μM SA were investigated. Addition of 10 μM SA did not significantly affect the culture and cytoplasm pH of both strains XC1 or Δ*rpfC* (Fig. 5). Exogenous addition of 50 or 100 μM SA also did not significantly affect culture and cytoplasmic pH at 12 hpi, although significant increases in both were observed at 24 and 36 hpi (Fig. 5). Further, the pH difference in the absence and presence of SA at 36 hpi was significantly higher than at 24 hpi (Fig. 5).

The culture and cytoplasmic pH of the SA-producing strains Δ*rpfC*::*pchAB* and XC1::*pchAB* at 12 hpi in XYS medium were similar to those of Δ*rpfC* and XC1 (Fig. 5). However, the culture and cytoplasmic pH of the strains Δ*rpfC*::*pchAB* and XC1::*pchAB* at 24 and 36 hpi were significantly higher than those of Δ*rpfC* and XC1 (Fig. 5).

To further investigate the roles of medium pH on the SA-dependent induction of BDSF and DSF turnover, XYS medium buffered at pH 7.0 (here, XYS-7.0) was prepared by adding KH_2_PO_4_-K_2_HPO_4_. The pH of Δ*rpfC* in XYS-7.0 remained around 7.0 during growth (Fig. S5A and B). Exogenous addition of 10 μM or 100 μM SA did not affect Δ*rpfC* growth and failed to induce BDSF and DSF turnover in Δ*rpfC* (Fig. S5A and C). Taken together, these results suggest that the SA-induced BDSF and DSF turnover is associated with the pH of culture medium.

10.1128/mbio.03644-21.5 FIG S5 SA has no effects on BDSF and DSF turnover in Δ*rpfC* growing in the XSY medium buffered at pH 7.0. (A) Growth of Δ*rpfC* in medium XYS (pH 7.0). (B) Culture pH of Δ*rpfC* in medium XYS (pH 7.0). (C) BDSF and DSF levels at 24 hpi in medium XYS (pH 7.0). Shown are the averages, along with standard deviations, for three technical repeats. Download FIG S5, TIF file, 0.6 MB. Copyright © 2022 Song et al. 2022 Song et al. https://creativecommons.org/licenses/by/4.0/ This content is distributed under the terms of the Creative Commons Attribution 4.0 International license.

### Increasing the pH of XYS medium triggers BDSF and DSF turnover in an RpfB-dependent manner.

**FIG 6:**
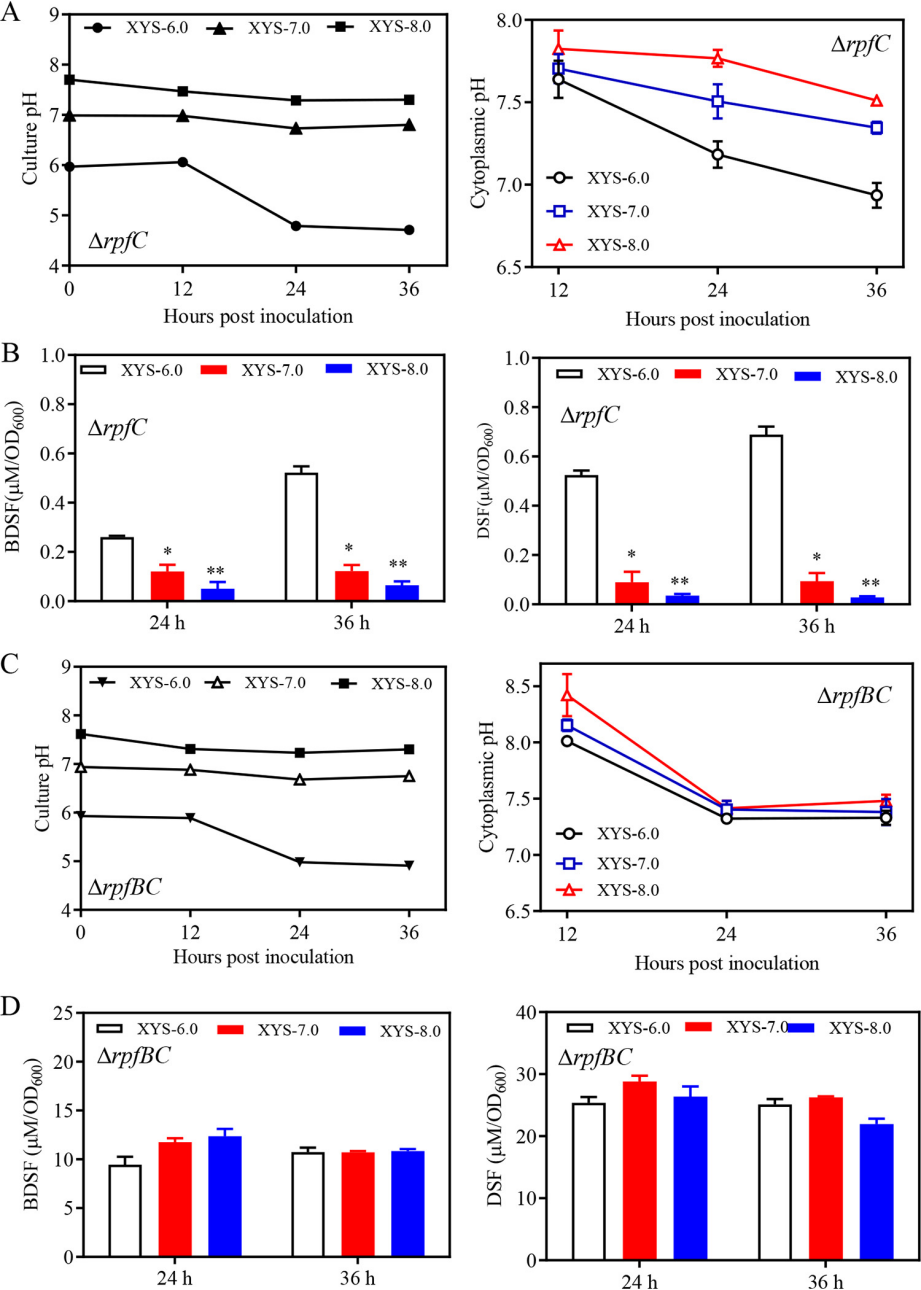
Increasing the culture pH induces DSF and BDSF turnover in an RpfB-dependent manner in Xanthomonas campestris pv. *campestris* strain. (A) The culture and cytoplasmic pH of Δ*rpfC* in the three XYS media pH buffered between 6.0 and 8.0, XYS-6.0, XYS-7.0, and XYS-8.0. (B) BDSF and DSF levels of Δ*rpfC* in the three XYS media at 24 hpi and 36 hpi. (C) The culture and cytoplasmic pH of *rpfC* and *rpfB* double deletion mutant Δ*rpfBC* in the three XYS media, XYS-6.0, XYS-7.0, and XYS-8.0. (D) BDSF and DSF levels in Δ*rpfBC* cultures at 24 and 36 hpi. Three independent experiments were conducted, and averages, along with standard deviations, are shown. Statistically significant differences are indicated (*, *P ≤ *0.05; **, *P ≤ *0.01).

To further verify whether the SA-induced culture and cytoplasmic pH increases resulted in BDSF and DSF turnover, three potassium phosphate-buffered XYS media formulations, including XYS-6.0 (pH 6.0), XYS-7.0 (pH 7.0), and XYS-8.0 (pH 8.0), were prepared. Δ*rpfC* exhibited similar growth patterns in all three medium types. The culture pH of Δ*rpfC* remained relatively stable in the XYS-7.0 and XYS-8.0 media but slowly declined from 6.06 at 12 hpi to 4.79 at 24 hpi in XYS-6.0, followed by a further decrease to 4.71 at 36 hpi (Fig. 6A). The cytoplasmic pH of Δ*rpfC* declined over time during growth in all three medium types (Fig. 6A). The BDSF and DSF levels of Δ*rpfC* cultures decreased as the medium pH increased from 6.0 to 8.0 (Fig. 6B). These results suggest that pH increases induce BDSF and DSF turnover.

The *rpfB* and *rpfC* double deletion mutant Δ*rpfBC* exhibited similar growth within the three types of media described above. The culture pH of Δ*rpfBC* remained relatively stable during growth in media XYS-7.0 and XYS-8.0, while the culture pH dropped from 5.9 at 12 hpi to a similar and constant value (4.9) at 24 hpi and 36 hpi in medium XYS-6.0 (Fig. 6C). The cytoplasmic pH of Δ*rpfBC* grown in the three types of media dropped from 8.1 to 8.3 at 12 hpi to a similar and constant value (7.4) at 24 hpi and 36 hpi in all three media (Fig. 6C). Further, no significant differences in BDSF and DSF levels for strain Δ*rpfBC* grown in the three medium types were observed (Fig. 6D).

### Establishment of an *in vitro* RpfB-dependent DSF turnover system.

**FIG 7:**
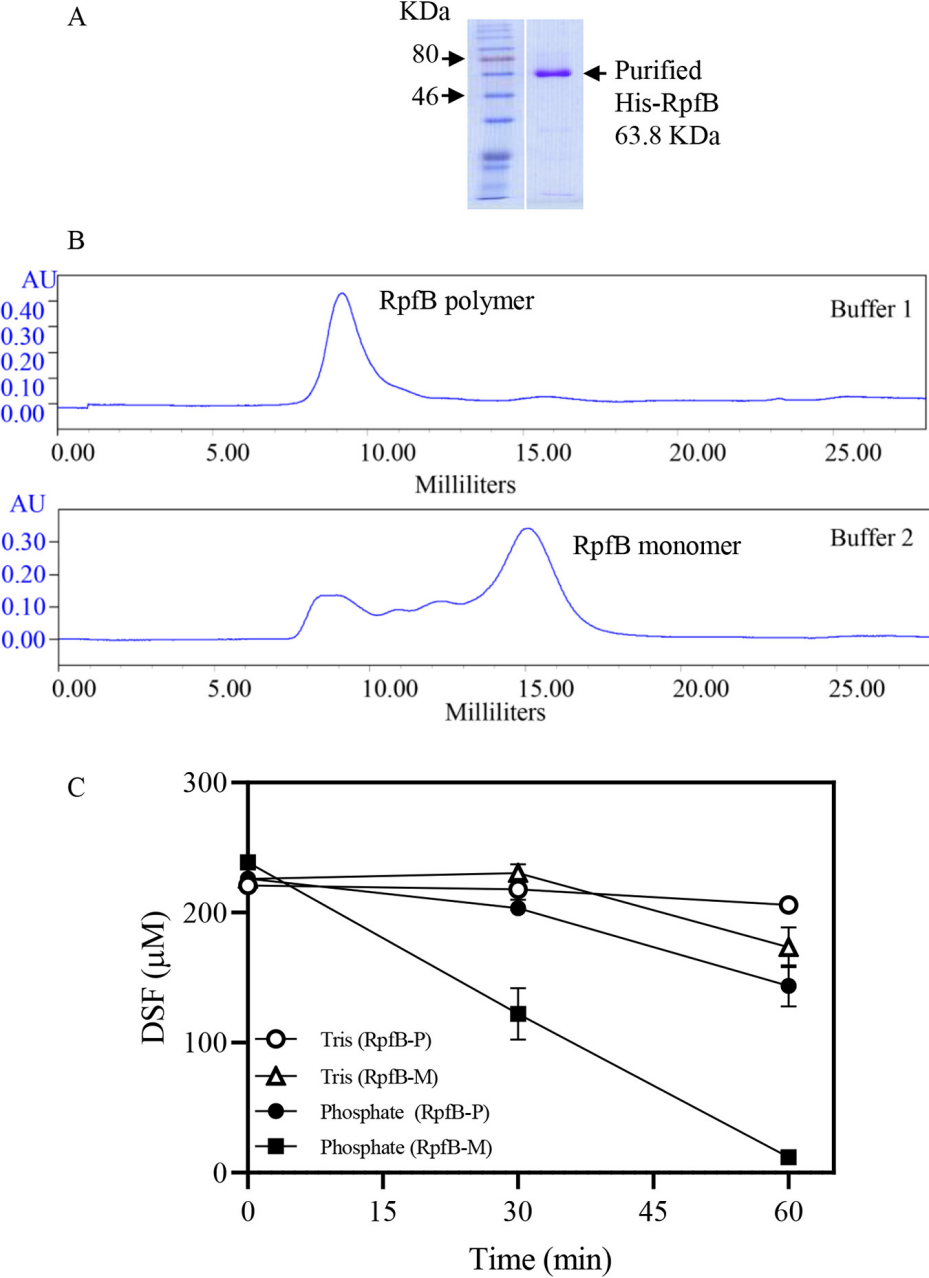
*In vitro* RpfB-dependent DSF turnover activity. (A) Purified His-tagged RpfB proteins. (B) Fast protein liquid chromatography (FPLC) analysis of purified RpfB protein, indicating the presence of monomer (RpfB-M) and polymer (RpfB-P) forms in elution buffers 1 and 2. Buffer 1 comprises 250 mM imidazole, 50 mM NaH_2_PO_4_, 300 mM NaCl, and 1 mM DTT (pH 7.4), while buffer 2 comprises 250 mM imidazole, 25 mM HEPES, 100 mM MgCl_2_, 100 mM NaCl, and 100 mM (NH_4_)_2_SO_4_ (pH 7.2). (C) *In vitro* DSF turnover activity of the RpfB monomers and polymers in Tris-based and phosphate-based buffers. Three independent experiments were conducted, and averages, along with standard deviations, are shown.

RpfB is one of the key enzymes required for DSF turnover (28). However, the *in vitro* DSF turnover activity of RpfB has not been demonstrated. Rather, RpfB was shown to only exhibit weak activity toward DSF in two previous studies (27, 28). In the present study, RpfB was expressed via the pET-28a vector, and His-tagged RpfB could be eluted from Ni-nitrilotriacetic acid (NTA) resins using the previously described elution buffer 1 (250 mM imidazole, 50 mM NaH_2_PO_4_, 300 mM NaCl, and 1 mM dithiothreitol [DTT], pH 7.4) and a HEPES- and magnesium-containing elution buffer 2 [250 mM imidazole, 25 mM HEPES, 100 mM MgCl_2,_ 100 mM NaCl, and 100 mM (NH_4_)_2_SO_4,_ pH 7.2] (Fig. 7A). Fast protein liquid chromatography (FPLC) assays revealed that most of the RpfB proteins eluted by buffer 1 were polymers (RpfB-P), while 71.2% of the total eluted RpfB proteins by buffer 2 were monomers (RpfB-M) (Fig. 7B).

The reaction mixture previously used for examining *in vitro* enzymatic activity of RpfB was a Tris-based buffer comprising 150 mM Tris-HCl, 10 mM MgCl_2_, 2 mM EDTA, 0.1% Triton X-100, 5 mM ATP, 0.5 mM CoA, 100 μM DSF, and 15 μg RpfB (pH 7.2) (27, 28). In our present study, both RpfB-P and RpfB-M exhibited very low enzymatic activity toward DSF in the Tris reaction mixture (Fig. 7B). A phosphate-containing reaction mixture was consequently developed by replacing the 150 mM Tris with 100 mM K_2_HPO_4_-KH_2_PO_4_. In the resultant phosphate reaction buffer, RpfB-P exhibited weak DSF turnover activity after 60 min of incubation, while RpfB-M exhibited strong DSF turnover activity (Fig. 7C). RpfB-M and the newly developed phosphate reaction buffer were subsequently used in the following RpfB enzymatic assays.

### *In vitro* RpfB-dependent DSF turnover activity increases with pH and is independent of SA.

**FIG 8:**
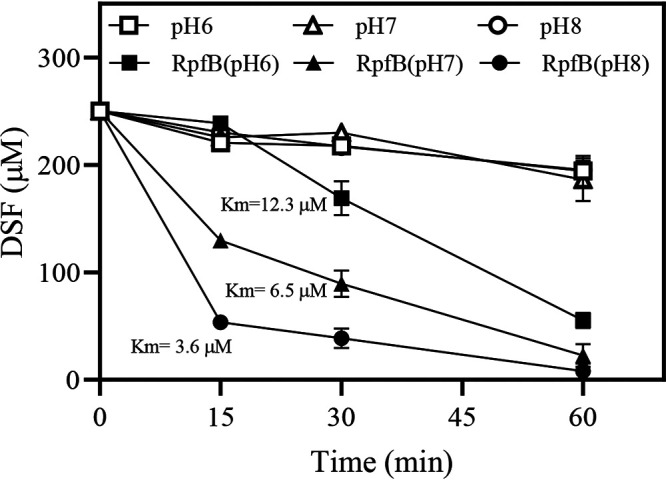
Increased pH in reaction mixtures significantly increases *in vitro* RpfB enzymatic activity for DSF. Phosphate reaction mixtures were buffered to pH of 6, 7, or 8 in the absence of RpfB (curves pH 6, pH 7, and pH 8) or in the presence of 10 μg of RpfB monomers (curves RpfB [pH 6], RpfB [pH 7], and RpfB [pH 8]). Michaelis constant (*K_m_*) values were determined by measuring the rate of catalysis at different DSF concentrations. Three independent experiments were conducted, and averages, along with standard deviations, are shown.

To investigate the effects of pH on RpfB enzymatic activity, three types of phosphate reaction mixtures with buffered pH of 6.0, 7.0, and 8.0 were prepared (referred to as pH 6, pH 7, and pH 8, respectively). DSF was then added at a final concentration of 250 mM, and the reaction mixtures were maintained at 37°C for 15, 30, and 60 min, respectively. In the absence of RpfB-M, there was no significant DSF turnover in the pH 6, pH 7, and pH 8 reaction mixtures (Fig. 8). In the presence of 10 μg RpfB-M, DSF levels decreased over time in all three reaction mixtures, including for RpfB (pH 6), RpfB (pH 7), and RpfB (pH 8) (Fig. 8). The calculated Michaelis constant (*K_m_*) values for RpfB enzymatic activity in the three reaction mixtures were 12.3 μM in RpfB (pH 6), 6.5 μM in RpfB (pH 7), and 3.6 μM in RpfB (pH 8) (Fig. 8).

To investigate whether the addition of SA to the phosphate reaction mixture can further enhance RpfB-dependent DSF turnover activity, SA was added to the DSF-containing reaction mixture at pH 6, 7, and 8 at a final concentration of 100 μM. After incubation at 37°C for 30 min, no significant differences in DSF levels were observed (Fig. S6), suggesting that *in vitro* RpfB-dependent DSF turnover activity is independent of SA.

10.1128/mbio.03644-21.6 FIG S6 Addition of SA to RpfB-containing reaction mixtures did not further increase DSF turnover activity *in vitro*. RpfB(−), reaction mixture containing no RpfB protein and SA; RpfB(+), reaction mixture containing RpfB protein but no SA; RpfB(+)SA, reaction mixture containing RpfB and SA. Three independent experiments were conducted, and averages, along with standard deviations, are shown. Download FIG S6, TIF file, 0.4 MB. Copyright © 2022 Song et al. 2022 Song et al. https://creativecommons.org/licenses/by/4.0/ This content is distributed under the terms of the Creative Commons Attribution 4.0 International license.

### SA-treated XC1 exhibits increased virulence in cabbage.

Xanthomonas campestris pv. *campestris* uses numerous virulence factors for pathogenicity and fitness in plant hosts (40). In this study, the production of DSF-regulated virulence factors in SA-treated XC1 (XC1+SA in this study) was investigated. Exogenous addition of SA (10 μM and 100 μM) had little effects on the production of extracellular polysaccharide (EPS) and extracellular protease in XYS plates or cultures (Fig. S7).

10.1128/mbio.03644-21.7 FIG S7 Extracellular polysaccharide production and extracellular protease activity of XC1 in the presence of SA. (A) EPS yield of XC1 in the presence of 10 to 100 μM SA. (B) Protease activity in the agar XYS plate supplemented with 2% milk powder. (C) Protease activity of XC1 in the XYS cultures supplemented with 10 to 100 μM SA. Download FIG S7, TIF file, 1.4 MB. Copyright © 2022 Song et al. 2022 Song et al. https://creativecommons.org/licenses/by/4.0/ This content is distributed under the terms of the Creative Commons Attribution 4.0 International license.

**FIG 9:**
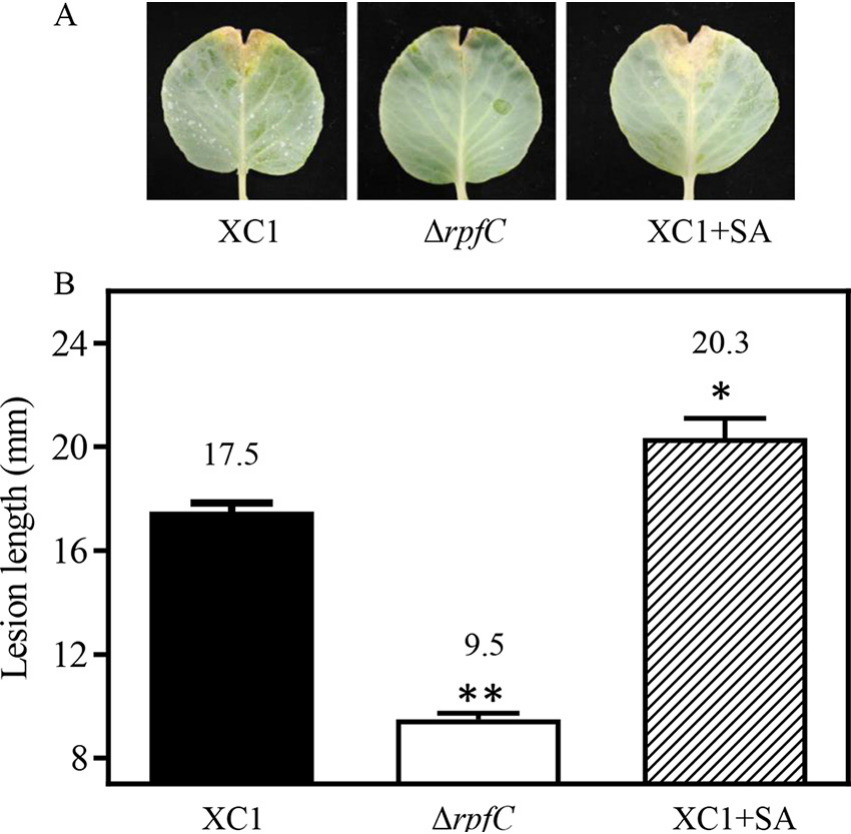
Virulence assays for Xanthomonas campestris pv. *campestris* strains in cabbage (Jingfeng-1). (A) Infection lesions at 14 days postinoculation. (B) Lesion lengths caused by strains XC1, Δ*rpfC*, and XC1 treated by SA (XC1+SA) at 14 days postinoculation in cabbage (Jingfeng-1). A total of 15 leaves were inoculated with each strain, and averages, along with standard deviations obtained, are shown. Statistically significant differences are indicated (*, *P ≤ *0.05; **, *P ≤ *0.01).

XC1 was treated with SA and then washed with PBS buffer to remove residual SA. The resultant strain XC1+SA, together with XC1 and Δ*rpfC*, were, respectively, inoculated into cabbage (Jingfeng-1) using the leaf-clipping method. Lesion lengths were then scored 2 weeks after inoculation. The control strain Δ*rpfC*, as expected, exhibited impaired virulence in cabbage compared with the wild-type strain XC1 (Fig. 9). Surprisingly, the strain XC1+SA exhibited 16% increment in lesion length compared with the wild-type strain XC1 (Fig. 9), suggesting that SA exposure increases Xanthomonas campestris pv. *campestris* virulence in cabbage.

## DISCUSSION

Plant colonization by phytopathogens is a very complex process in which a variety of factors are involved. SA is considered the “sixth” phytohormone that is produced via two independent pathways (e.g., through isochorismate synthase and phenylalanine ammonia lyase) in plants (5, 41). Most plants maintain relatively low SA levels during normal growth and development. However, a rapid increase in localized endogenous SA levels (i.e., millimolar levels) occurs after pathogen infections (42, 43). SA functions within plants to trigger pathogen-associated molecular pattern (PAMP)-triggered immunity (PTI)-, effector-triggered immunity (ETI)-, and systemic acquired resistance (SAR)-dependent defenses against phytopathogens (3, 5, 11). On the other hand, DSF-dependent QS is a cell density-dependent mechanism for the pathogen Xanthomonas campestris pv. *campestris* to produce extracellular enzymes and other virulence factors at all stages of disease development (17, 18, 20, 44). Despite that SA signaling within host plants and DSF signaling in the pathogen Xanthomonas campestris pv. *campestris* are well studied, the present study demonstrated, for the first time, that SA produced by the host plant can also directly act on the QS system of the invading Xanthomonas campestris pv. *campestris* pathogen to induce DSF turnover via RpfB in a pH-dependent manner, thereby ultimately affecting QS-dependent virulence.

SA has been previously reported to reduce *N*-acyl-homoserine lactone-type QS signal biosynthesis at the transcriptional level in the plant pathogens P. carotovorum and Pectobacterium aroidearum. In the absence of SA, the relative expression of *expI* and *expR* in strain P. carotovorum PC1 increased with time and increased by about 2.5- and 10.0-fold after 24 h of growth. In the presence of SA, no such time-dependent increases in the expression of these genes were observed (11). In A. tumefaciens C58, SA exposure (6 μM for 6 h) stimulated the expression of the *attKLM* operon that encodes lactonase, which can hydrolyze QS signal molecules, namely, *N*-3-oxo-octanoyl-homoserine lactone (3OC8-HSL) in A. tumefaciens (6). In the present study, exogenous application of SA or endogenous production of SA significantly reduced the levels of the QS signals BDSF and DSF at 24 and 36 hpi during growth in XYS medium (Fig. 2 and 3). However, exogenous addition of SA at 10- to 100-μM levels did not affect the transcriptional levels of *rpfF* and *rpfB*, encoding two key enzymes that are responsible for DSF biosynthesis and turnover in Xanthomonas campestris pv. *campestris* (21, 28) (Fig. S3). Thus, the SA-dependent BDSF and DSF turnover in Xanthomonas campestris pv. *campestris* does not occur at the transcriptional level but, rather, occurs via a pH-dependent activation of RpfB. Compared with the SA-induced QS signal turnover that occurs at the transcriptional level in P. carotovorum, P. aroidearum, and A. tumefaciens (6, 11), this mechanism might provide a faster and more efficient means for host plants to turn over the DSF signals of invading pathogens.

To elucidate the molecular mechanisms underlying SA-dependent induction of BDSF and DSF turnover, environmental conditions leading to this result were investigated. SA-induced BDSF and DSF turnover was found to only occur at 24 hpi and 36 hpi in XYS medium (Fig. 2; Fig. 5) but not in rich media NYG and NA (data not shown). Further results showed that XC1 and Δ*rpfC* growth in XYS medium led to an acidic extracellular pH at 24 hpi and 36 hpi, respectively (Fig. 5). The intracellular pH of XC1 and Δ*rpfC* grown in medium XYS was alkaline but declined during growth (Fig. 5). Interestingly, SA-induced *attKLM* expression and 3OC8-HSL turnover in the plant pathogen A. tumefaciens were also found to only occur under acidic conditions (6). The acidic extracellular pH- and growth stage-dependent SA induction of DSF turnover is probably associated with the acidic extracellular pH-dependent SA uptake and the cell density-dependent RpfB expression pattern. First, the increased uptake of SA at acidic pH was observed in Escherichia coli (45). Whether a similar mechanism occurs in Xanthomonas campestris pv. *campestris* requires further investigation. Second, RpfB was demonstrated to be a fatty acyl-CoA ligase to effectively turn over DSF family signals via the β-oxidation pathway in Xanthomonas campestris pv. *campestris* and Xanthomonas oryzae pv. oryzae (Xoo). The expression of *rpfB* is significantly enhanced when *Xanthomonas* cells enter the stationary phase (21, 29). Further, the *in vitro* conditions (XYS medium and acidic extracellular pH) for SA induction of BDSF and DSF turnover are highly consistent with the *in planta* conditions encountered by Xanthomonas campestris pv. *campestris* during infection of host plants. Specifically, Xanthomonas campestris pv. *campestris* is typically restricted to the xylem vessels of infected plants during the late stages of disease development (46). The XYS medium composition, containing basic inorganic salts, 5 g L^−1^ sucrose, and 0.0625% yeast extract, was thought to be similar to the natural nutrient conditions encountered by Xanthomonas campestris pv. *campestris* inside plant xylem (21, 47). Further, the apoplastic and vascular pH of plants is acidic (48, 49). Finally, both SA-treated and SA-producing Xanthomonas campestris pv. *campestris* strains exhibited increased virulence in host plant cabbage (Fig. 9). Therefore, these observations support the supposition that SA plays a role in the induction of QS signal turnover during Xanthomonas campestris pv. *campestris* infection.

**FIG 10:**
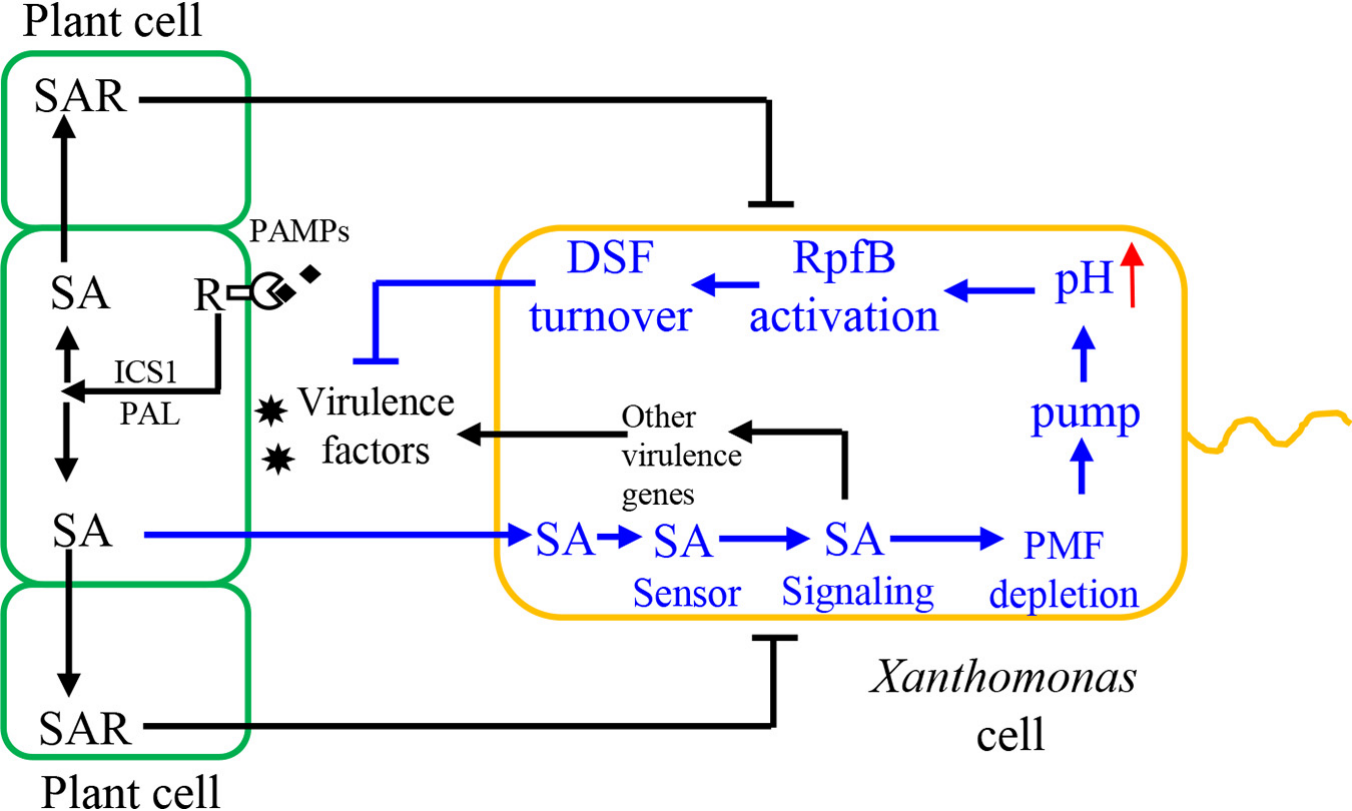
Proposed model showing the molecular interactions between host plants and *Xanthomonas* pathogens via SA signaling. The Xanthomonas campestris pv. *campestris*-derived pathogen-associated molecular patterns (PAMPs) are sensed by the receptor (R) proteins located in plant cell membrane; in turn, the isochorismate synthase 1 (ICS1)- and phenylalanine ammonia lyase (PAL)-dependent SA biosynthetic pathways are activated. SA is transported from infected plant cell into distal cell to induce systemic acquired resistance (SAR) to counter with Xanthomonas campestris pv. *campestris* infection. SA is permeated or transported into Xanthomonas campestris pv. *campestris* cell and sensed by a putative SA sensor. The subsequent SA signaling pathway alters the proton motive force (PMF) in Xanthomonas campestris pv. *campestris*, which further activates the related efflux pumps to lead to a culture and cytoplasmic pH increase. Increment of pH activates RpfB enzymatic activity for DSF and BDSF turnover, which negatively regulates virulence factor production. SA signaling also positively regulates virulence factor production via an unknown pathway. Upward red arrow indicates increased pH. T-arrow indicates the inhibitory effects of host cell SAR on *Xanthomonas* infection.

To further clarify the molecular mechanism underlying the SA-induced BDSF and DSF turnover, the relationships among SA, pH, and RpfB enzymatic activity were investigated. Exogenous addition of SA or endogenous production of SA significantly increased the culture and cytoplasmic pH at 24 and 36 hpi in XYS medium (Fig. 5). In the absence of SA, increasing culture pH of the XYS medium significantly induced BDSF and DSF turnover (Fig. 6). These results suggest that the SA-induced increase in pH is associated with BDSF and DSF turnover. Further, the *in vitro* assay of RpfB-dependent DSF turnover activity was established (Fig. 7). Increasing the pH of the reaction mixture from 6 to 8 significantly increased the *in vitro* activity of RpfB toward DSF turnover (Fig. 8). Furthermore, addition of SA to the RpfB-containing reaction mixture failed to further increase DSF turnover activity (Fig. S6 in the supplemental material), suggesting that SA probably cannot directly interact or bind to RpfB to alter its enzymatic activity. Taken together, these results support the hypothesis that exogenous addition of SA or endogenous production of SA leads to increased culture and cytoplasmic pH. The increased cytoplasmic pH, in turn, increases the enzymatic activity of RpfB, which finally leads to DSF turnover (Fig. 10).

To discern how SA causes increased pH in Xanthomonas campestris pv. *campestris* XYS cultures, SA sensing and signaling in Xanthomonas campestris pv. *campestris* are needed in future studies. The MarR family of transcription factor SlyA has been well characterized in Salmonella enterica serovar Typhimurium, where it serves primarily to upregulate virulence genes. The addition of 25 mM or 50 mM SA led to complete dissociation of SlyA from the target DNA, suggesting that SA could be an effector for SlyA at high concentrations (50). Further determination of the SA-SlyA cocrystal structure revealed that SA binds and stabilizes the SlyA dimer in a conformation unfavorable for DNA binding (51). Notably, SlyA homolog is present in Xanthomonas campestris pv. *campestris*. The presence of the SA sensor and SA signaling pathway in Xanthomonas campestris pv. *campestris* is under investigation and will provide additional insights into the regulation of genes via SA.

SA is a membrane-permeant aromatic acid. In E. coli, SA has been shown to induce a drug resistance regulon but to deplete proton motive force (PMF). The PMF drives the efflux pump MdtEF-TolC and related pumps, which are required for adaptation for transient extreme acid exposure (45). In plants, apoplastic pH increases have also been shown to occur during plant-pathogen interactions and in response to saline or drought stresses (49). Changes in apoplastic pH can be achieved by modulating the activities of H^+^-extruding ATPases, via H^+^-coupled nutrient transporters in the plasma membrane, and by exporting acid metabolites (48). Further characterization of these activities in SA-treated Xanthomonas campestris pv. *campestris* cells might provide additional insights into the underlying mechanisms involved in SA-dependent pH increase and DSF turnover.

The QS signal DSF has been shown to positively regulate the production of EPS and protease in XC1 (52). The present study showed that SA could induce DSF turnover; however, no significant reduction in the levels of EPS and protease was observed in SA-treated XC1 (Fig. S7). Instead, SA-treated XC1 was found to be more virulent than the wild-type strain XC1 in cabbage (Fig. 9). Further transcriptome sequencing (RNA-Seq) analysis of the SA-treated XC1 revealed that SA also positively regulates several virulence-associated genes and gene clusters. The increased virulence of SA-treated XC1 probably resulted from the combined effects of these differential SA-regulated biological functions.

In summary, the results of this study strongly support the hypothesis that the plant defense signal SA induces gene expression changes within plants but also directly acts on plant pathogens, with QS machinery being a particularly important target of SA modulation. SA apparently induces DSF signal turnover via a medium- and pH-dependent manner, ultimately resulting in increased RpfB activity that leads to higher turnover of DSF and BDSF. Additional research is required to determine how SA induces pH increases inside Xanthomonas campestris pv. *campestris* cells and whether SA asserts additional changes on Xanthomonas campestris pv. *campestris*. Insights into these areas could hold significant potential for the development of control measures against *Xanthomonas* pathogens. DSF signaling and RpfB-dependent DSF turnover are also present in a range of human opportunistic pathogens, such as Stenotrophomonas maltophilia and *Burkholderia* spp., and the important environmental microbes such as *Lysobacter*, *Leptospirillum*, *Frateuria*, *Luteibacter*, *Rhodanobacter*, Methylobacillus flagellates, and Thiobacillus denitrificans; therefore, future research on these bacteria could also benefit from this finding.

## MATERIALS AND METHODS

### Bacterial strains and culture conditions.

The bacterial strains and plasmids used in the present study are shown in Table S1 in the supplemental material. Xanthomonas campestris pv. *campestris* wild-type strain XC1 and its derivatives were grown at 28°C in XYS medium (0.7 g L^−1^ K_2_HPO_4_, 0.2 g L^−1^ KH_2_PO_4_, 1 g L^−1^ (NH_4_)_2_SO_4_, 0.1 g L^−1^ MgCl_2_·6H_2_O, 0.01 g L^−1^ FeSO_4_·7H_2_O, 0.001 g L^−1^ MnCl_2_·4H_2_O, 5 g L^−1^ sucrose, and 0.0625% yeast extract, pH 7.0), NYG medium (5 g L^−1^ peptone, 3 g L^−1^ yeast extract, and 20 g L^−1^ glycerol), Luria-Bertani (LB) medium (5 g L^−1^ yeast extract, 10 g L^−1^ peptone, and 10 g L^−1^ sodium chloride), or nutrient agar (NA) medium (5 g L^−1^, 3 g L^−1^ beef extract, 10 g L^−1^ sucrose, and 1 g L^−1^ yeast extract, pH 7.0). Tryptone, peptone, beef extract, and yeast extract were purchased from Sangon Biotech (Shanghai, China). E. coli DH5α cells were used as hosts for constructing all recombinant vectors. E. coli strains were cultured at 37°C in LB medium. Antibiotics were then added at the following concentrations when needed: 25 μg mL^−1^ rifamycin (Rif), 50 μg mL^−1^ kanamycin (Km), 20 μg mL^−1^ gentamicin (Gm), and 100 μg mL^−1^ ampicillin (Amp). Bacterial growth was determined by measuring optical density at a wavelength of 600 nm (OD_600_).

10.1128/mbio.03644-21.8 TABLE S1 Bacterial strains, plasmids, and oligonucleotides used in this study. Download Table S1, DOCX file, 0.02 MB. Copyright © 2022 Song et al. 2022 Song et al. https://creativecommons.org/licenses/by/4.0/ This content is distributed under the terms of the Creative Commons Attribution 4.0 International license.

### Gene deletion and functional complementation analysis in Xanthomonas campestris pv. *campestris*.

An in-frame gene deletion was conducted using previously described methods (23). Briefly, the flanking fragments (∼500 bp) of the target deletion region were fused using overlap extension PCR. The resultant fragment was then subcloned into the suicide vector pK18mobsacB. The resultant recombinant plasmid was introduced into Xanthomonas campestris pv. *campestris* strains and then subsequently integrated within the target region via homologous recombination. The resultant strain was then plated on NYG agar plates with 50 μg mL^−1^ Rif and 5% (wt/vol) sucrose to allow a second single-crossover homologous recombination event resulting in allelic exchange. Gene deletion was verified by colony PCR and subsequent DNA sequencing. For complementation analysis, the target gene was PCR amplified and cloned into the multiple cloning site of the versatile delivery vector mini-Tn*7*T-Gm. The resultant constructs were then transferred into Xanthomonas campestris pv. *campestris* strains following previously described methods (53). The primers used for this process are shown in Table S1.

### Extraction and quantitative analyses of SA in Xanthomonas campestris pv. *campestris* cultures and in plant leaf tissues.

The extraction and purification of salicylic acid in XC1 cultures were performed following previously described methods (33). SA production levels were then quantified by high-performance liquid chromatography (HPLC), following previously described methods (33). Briefly, 0.5 mL of the XC1 strain culture supernatant and its derived strains were collected and adjusted to pH 4.0 for extraction with 1 mL of ethyl acetate. The ethyl acetate fractions were then collected and evaporated, followed by dissolving in 0.1 mL of methanol for HPLC analysis with a C_18_ reverse-phase column (Zorbax XDB; 5 μm, 4.6 by 150 mm). Fractions were then eluted with methanol and water containing 0.05% formic acid in each phase (25:75 [vol/vol]) at 1 mL min^−1^. Commercially available SA (Sigma, USA) was used as a standard.

Cabbage (Jingfeng-1) was grown in a light incubator at 25°C and 75% humidity with a photoperiod of 16 h (8,000 lx) for 2 months. The mature leaves were inoculated with Xanthomonas campestris pv. *campestris* cultures at an OD_600_ of 0.1 or with phosphate-buffered saline (PBS; 1×, pH 7.4) using the leaf-clipping method and then maintained at 28°C and 90% humidity with a photoperiod of 16 h (8,000 lx). Leaf discs next to the inoculation sites (200 mg) were collected at 5, 9, and 16 days postinoculation, followed by extraction of SA from the leaf tissues according to previously described methods (54). The extract was finally dissolved in 50 μL of methanol. HPLC coupled with triple-quadrupole tandem mass spectrometry (HPLC-QqQ-MS/MS) (Agilent, USA) was used for quantitative analysis of SA concentrations in leaf tissues (34). Briefly, a total of 10 μL of extract was loaded into the Zorbax Eclipse XDB C_18_ column (4.6 by 150 mm, 5 μm; Agilent) for chromatographic separation. The column was then eluted with methanol and water with 0.1% formic acid in each phase (60/40 [vol/vol]) over 40 min at a flow rate of 0.4 mL min^−1^. A triple-quadrupole tandem mass spectrometer equipped with an electrospray ion source (ESI) (Agilent, USA) was used for quantitative analysis. The multiple-reaction monitoring (MRM) mode was used to record MS spectra. An Agilent optimizer software program was then used to optimize the fragmentation, collision energy, precursor ion screening, and product ion screening. SA concentration in the cabbage leaves is expressed as concentration per fresh weight (FW) of starting vegetal tissue materials.

### Extraction, purification, and quantitative analysis of BDSF and DSF using UPLC-TOF MS.

Extraction and quantitative analysis of DSF and BDSF in Xanthomonas campestris pv. *campestris* cultures were performed following previously described methods (55). Briefly, 20 mL of liquid cultures were collected for each strain. Cultures were then adjusted to pH 4.0 prior to extraction with 20 mL of ethyl acetate. Ethyl acetate was then removed by rotary evaporation at 30°C. The residues were subsequently dissolved in 0.1 mL of methanol and analyzed using ultrahigh-performance liquid chromatography–time of flight mass spectrometry (UPLC-TOF MS) (Agilent, USA) with a C_18_ reverse-phase column (Zorbax XDB; 5 μm, 4.6 by 150 mm) (Agilent, USA). Methanol and water containing 0.1% formic acid (80/20 [vol/vol]) were used to elute the sample at a rate of 0.4 mL min^−1^.

### RpfB expression, purification, and *in vitro* DSF turnover activity assays.

E. coli strain BL21(DE3) cells containing the expression vector pET28a-RpfB were used as previously described (28). RpfB protein expression was induced by adding 0.1 mM IPTG (isopropyl-β-d-thiogalactopyranoside) and growing at 18°C for 16 h. Bacterial cells were collected by centrifugation and resuspended in the recommended lysis buffer 1 and a modified lysis buffer 2 containing 25 mM HEPES, 100 mM NaCl, 100 mM MgCl_2_·6H_2_O, 100 mM (NH_4_)_2_SO_4_, and 10 mM imidazole (pH 7.2). The recombinant His-tagged RpfB protein was then purified using a nickel-ion affinity column (Smart-Lifesciences). The column was washed with a modified wash buffer containing 25 mM HEPES, 100 mM NaCl, 100 mM MgCl_2_·6H_2_O, 100 mM (NH_4_)_2_SO_4_, and 25 mM imidazole (pH 7.2). His-tagged RpfB proteins were eluted in a modified elution buffer [25 mM HEPES, 100 mM NaCl, 100 mM MgCl_2_·6H_2_O, 100 mM (NH_4_)_2_SO_4_, and 250 mM imidazole, pH 7.2]. The eluted RpfB-His protein was then loaded into a Superdex 200 gel filtration column (GE Healthcare) equilibrated with a nonimidazole buffer containing 25 mM HEPES, 100 mM NaCl, 100 mM MgCl_2_·6H_2_O, and 100 mM (NH_4_)_2_SO_4_ (pH 7.2).

*In vitro* DSF turnover assays were conducted following previously described methods with slight modifications (28). Specifically, the Tris buffer in the reaction mixture was replaced with a phosphate buffer. The modified reaction mixture contained 100 mM K_2_HPO_4_-KH_2_PO_4_, 10 mM MgCl_2_, 2 mM EDTA, 0.1% (vol/vol) Triton X-100, 5 mM ATP, 0.5 mM reduced CoA, and 0.3 mM DSF in addition to 10 μg of purified His-tagged RpfB or heat-inactivated His-tagged RpfB in a total volume of 500 μL. Reaction mixtures were maintained at 28°C. After inoculation for 15, 30, 45, and 60 min, the pH of the reaction mixture was adjusted to pH 4.0 by addition of 1 M HCl prior to extraction with a 2-fold volume of ethyl acetate. Quantitative analysis of DSF in the reaction mixture was then performed as described above.

### Construction of *gusA*-dependent reporter strains to monitor *rpfF* and *rpfB* transcriptional activities and GUS assays.

The construction of *gusA*-based reporter strains for *rpfF* and *rpfB* was performed as previously described (28). Quantitative β-glucuronidase (GUS) activity assays were also performed as previously described (34). Briefly, the reporter strains were grown in XYS medium for 12 h to 36 h at 28°C. A sample of culture (1 mL) was then collected, centrifuged at 8,000 rpm (Thermo Scientific; Legend Micro 17R) for 10 min, and washed once with PBS buffer (1×, pH 7.4). Bacterial cells were then suspended in 1 mL PBS buffer following the addition of 20 μL of 0.1% (wt/vol) SDS solution and 40 μL of chloroform. Centrifugation was then conducted at 8,000 rpm for 5 min, and 100 μL of supernatant was removed and added to 250 μL of MUG solution (1 mM 4-methylumbelliferyl β-d-glucuronide, 50 mM PBS, 5 mM DTT, and 1 mM EDTA, pH 8.0). The reaction mixtures were subsequently incubated at 37°C for 15 min. Each reaction (200 μL) was terminated by mixing with 800 μL of 0.2 M Na_2_CO_3_ solution. A 96-well plate was used to detect GUS activities with a fluorescence microplate reader based on excitation and emission wavelengths of 365 nm and 455 nm, respectively.

### Western blotting.

Proteins were separated by SDS-PAGE and electrotransferred onto a polyvinylidene difluoride (PVDF) membrane (Roche, USA). After blocking with 5% (wt/vol) nonfat milk powder, the membranes were incubated with the 1:5,000 diluted polyclonal antibodies against RpfF or RpfB (28). The membrane was subsequently washed four times with TBST buffer (20 mM Tris, 0.15 M NaCl, and 0.1% [vol/vol] Tween 20). Then, a 1:6,500 diluted horseradish peroxidase (HRP)-conjugated goat anti-rabbit IgG (Abmart; catalog no. M21001) was used as secondary antibody. After washing the membrane four times, the luminescent signal was detected with an ECL kit and a ChampChemi 610 Plus instrument (Sage Creation Science, China).

### Measurement of culture and cytoplasmic pH.

A total of 3 mL of Xanthomonas campestris pv. *campestris* cultures was collected and centrifuged at 5,000 rpm for 5 min at 4°C for subsequent pH value determination of cytoplasmic and Xanthomonas campestris pv. *campestris* culture. The pH of the resultant supernatants was measured as culture pH using a pH meter. The cytoplasmic pH of Xanthomonas campestris pv. *campestris* strain cells was measured following methods described by Zarkan et al. (38). Briefly, genes that encoded the mCherry protein (GenBank accession no. AY533296) and pHluorin (GenBank accession no. AY678264) were synthesized in Sangon, Shanghai. The fusion gene encoding an mCherry and pHluorin translational fusion protein via a (Gly-Gly-Ser)_2_ linker was generated by PCR. The resultant fusion gene was then cloned into the expression vector pBBR1MCS-2 to generate the recombinant plasmid pBBR-pHluorin-mCherry. The resultant plasmid was then transformed into Xanthomonas campestris pv. *campestris* strain cells by triparental mating. Primers used in this process are shown in Table S1.

A fluorescence microplate reader was used to measure the fluorescence emission intensity of pHluorin (excitation and emission, 488 and 510 nm, respectively) and mCherry (excitation and emission, 587 and 610 nm, respectively). Samples were taken every 12 h during cultivation. Cytoplasmic pH was then calculated from the intensity ratio of pHluorin to mCherry using a standard curve. To establish the standard curve, four Xanthomonas campestris pv. *campestris* culture samples at an OD_600_ of 0.6 were collected, and the culture medium pH was manually adjusted to 6.0, 7.0, 7.5, and 8.0 using 6 M HCl, followed by addition of 250 μM carbonyl cyanide *m*-chlorophenyl hydrazone, resulting in a similar cytoplasmic pH to that of the media. Cells were left for 20 min before measuring the intensity of pHluorin and mCherry in the fluorescence microplate reader.

### Virulence assay of Xanthomonas campestris pv. *campestris* strains in cabbage.

The virulence of Xanthomonas campestris pv. *campestris* strains in cabbage (Jingfeng-1) was estimated by leaf clipping as previously described (34). Briefly, the XC1 and Δ*rpfC* strains were grown in XYS liquid medium for 12 h and then resuspended in PBS buffer at a final OD_600_ of 0.1. SA-treated XC1 (XC1+SA in this study) was prepared by growing wild-type XC1 in XYS liquid medium supplemented with 100 μM SA for 12 h. Cells were then collected by centrifugation at 5,000 rpm for 5 min at 4°C. The resultant cell pellet was then washed using PBS buffer twice to remove the residual SA and resuspended in PBS buffer at a final OD_600_ of 0.1. Leaves were then cut with sterile scissors dipped in bacterial suspensions with OD_600_ values of 0.1. After inoculation, the infected plants were wrapped with transparent plastic film and maintained in a growth chamber at 30°C with a relative humidity of 80% and a light intensity of 10,000 lx. Lesion length was measured 2 weeks after inoculation. A total of 15 leaves were inoculated with each strain, and the average lesion lengths with standard deviation (SD) values are shown.

### Statistical analyses.

All experiments were performed at least in triplicate. Analysis of variance (ANOVA) tests for all experimental data sets were performed using the JMP software program (version 5.0). Significant effects of all treatments were determined by *F* values. When a significant F test was observed, separation of means analysis was accomplished with Fisher’s protected least significant difference test using a *P* value of <0.05.
