# Supplementary figures and images for: The Plant Defense Signal Salicylic Acid Activates the RpfB-Dependent Quorum Sensing Signal Turnover via Altering the Culture and Cytoplasmic pH in the Phytopathogen Xanthomonas campestris
Source: mBio. 2022 Mar 7;13(2):e03644-21. doi: 10.1128/mbio.03644-21 (PMC9040794; doi:10.1128/mbio.03644-21)

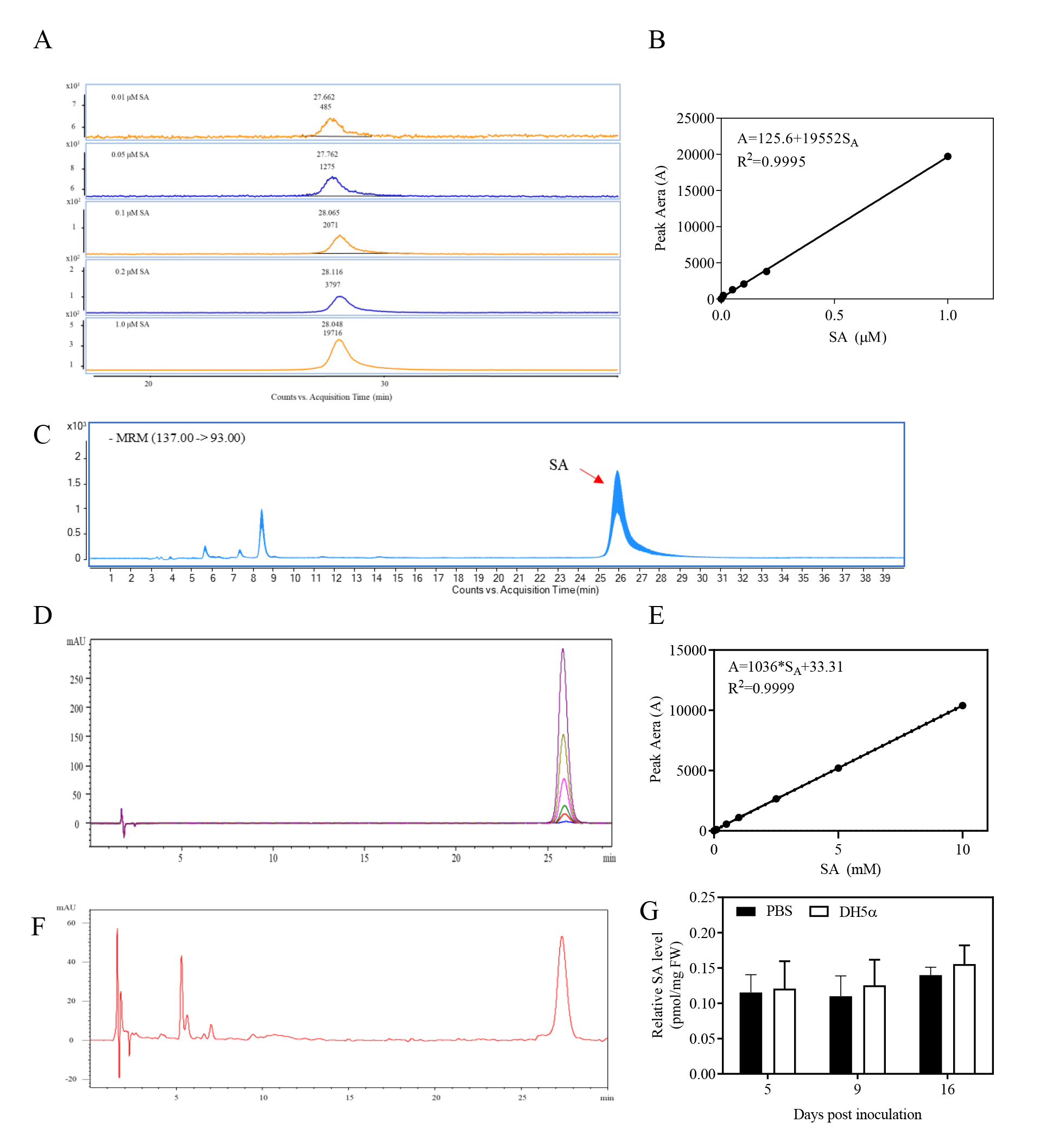

Supplement: FIG S1 [file mbio.03644-21-sf001.tif]

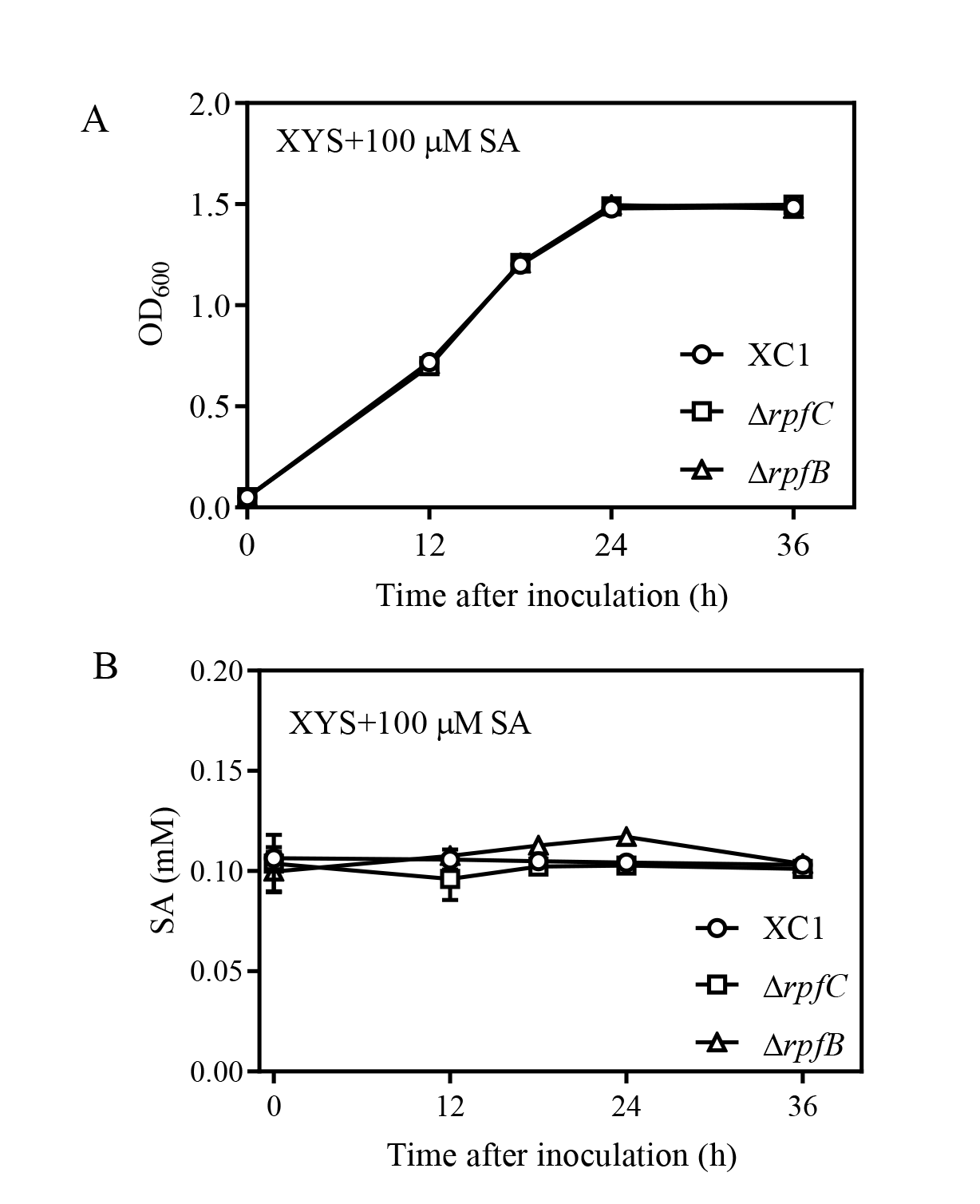

Supplement: FIG S2 [file mbio.03644-21-sf002.tif]

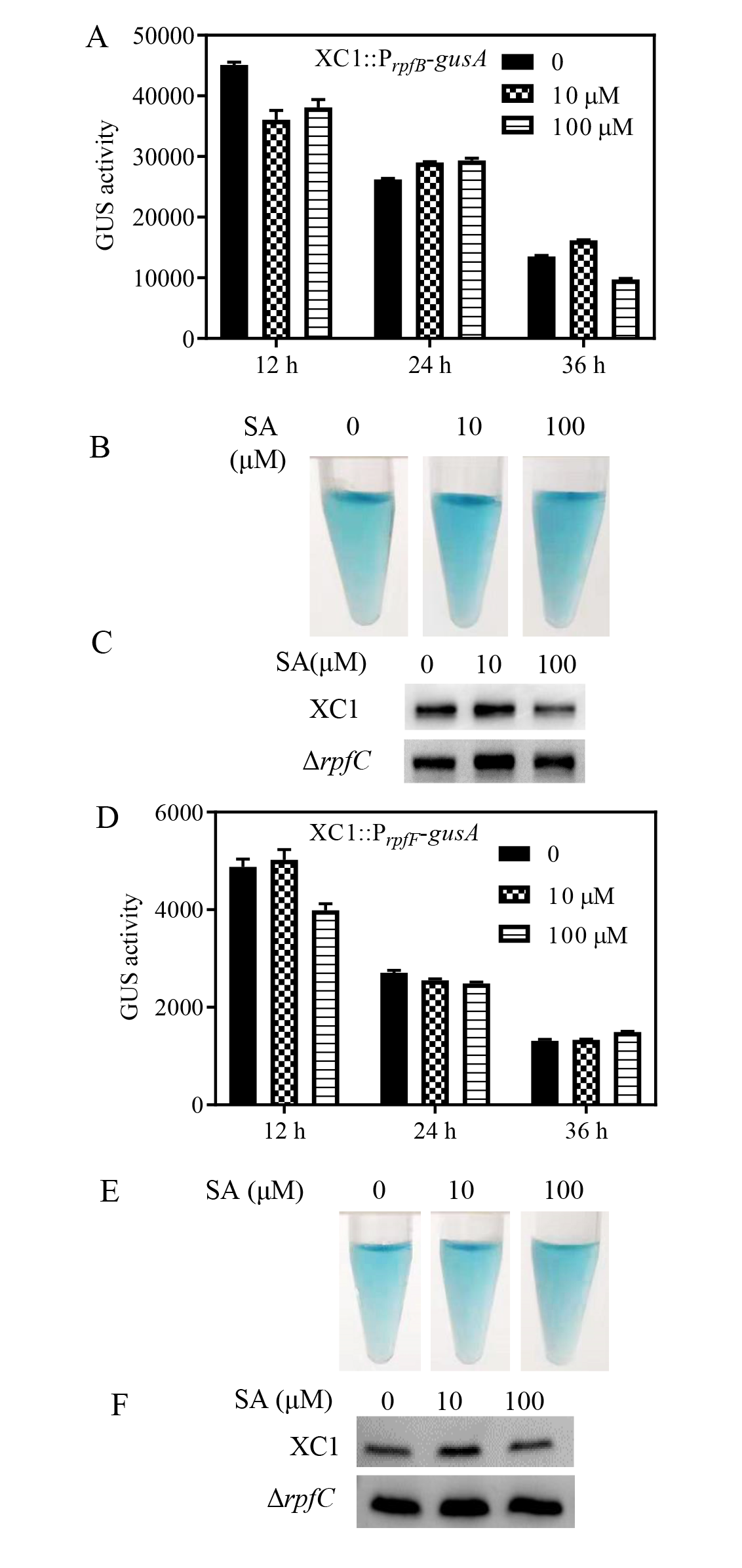

Supplement: FIG S3 [file mbio.03644-21-sf003.tif]

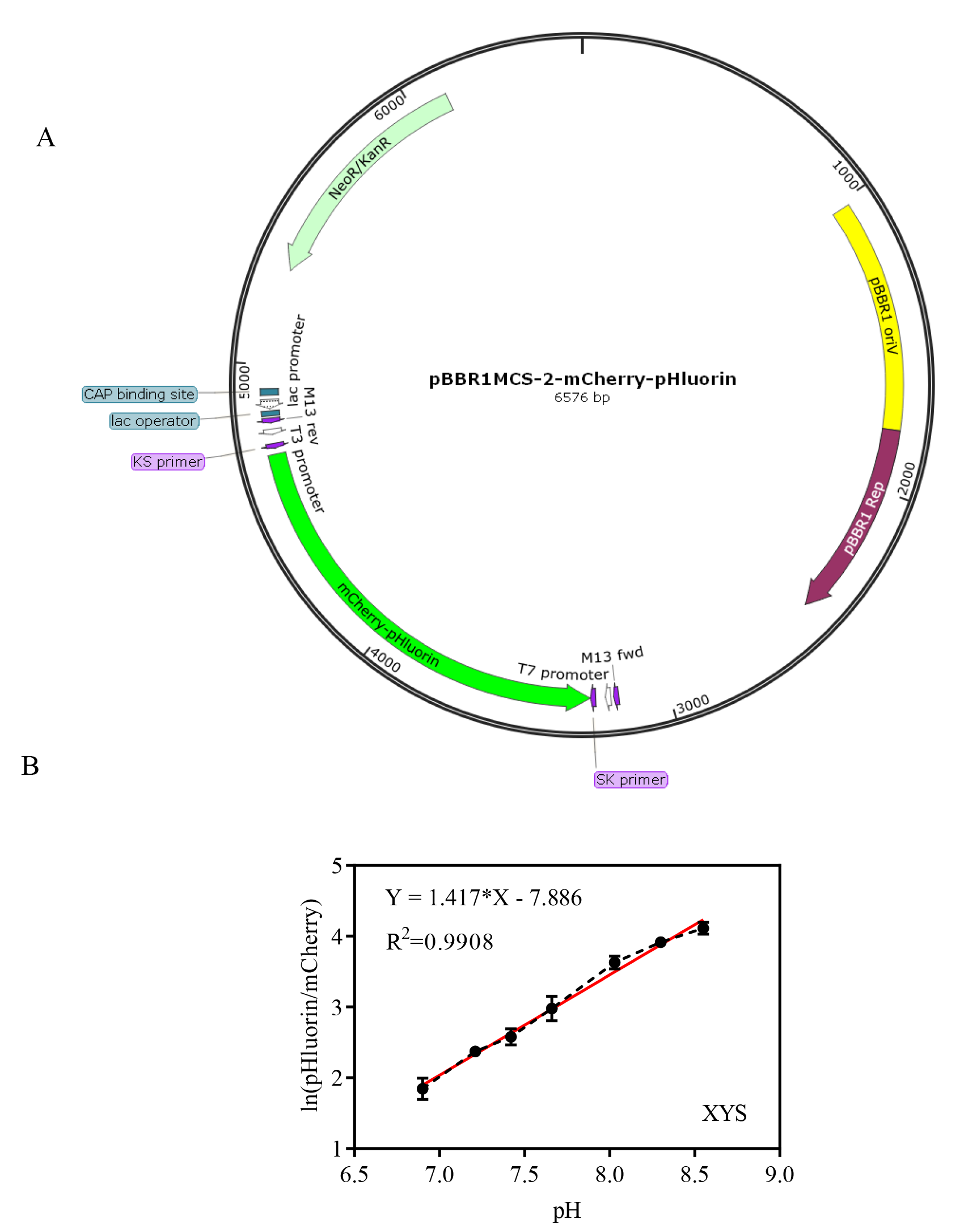

Supplement: FIG S4 [file mbio.03644-21-sf004.tif]

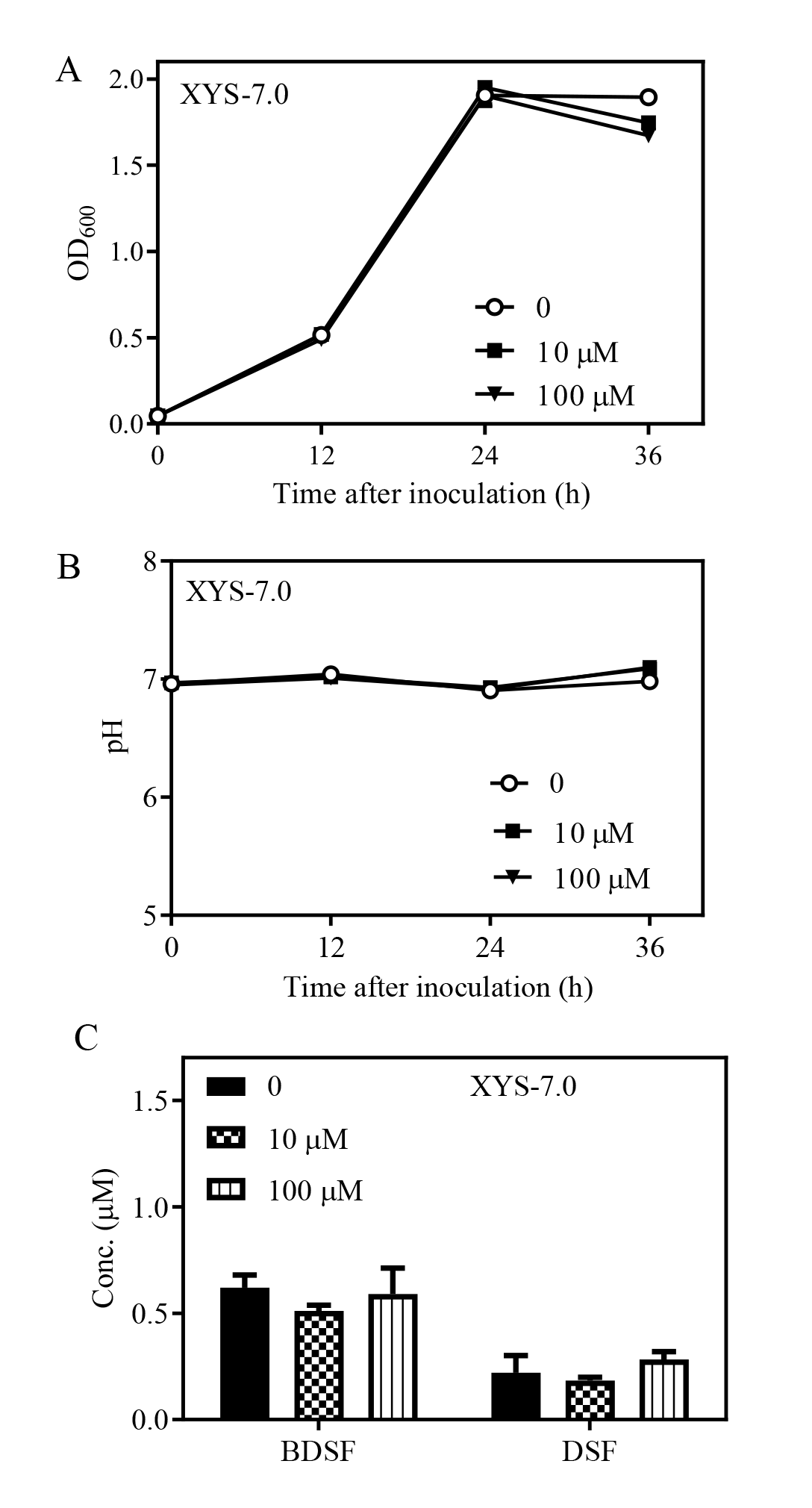

Supplement: FIG S5 [file mbio.03644-21-sf005.tif]

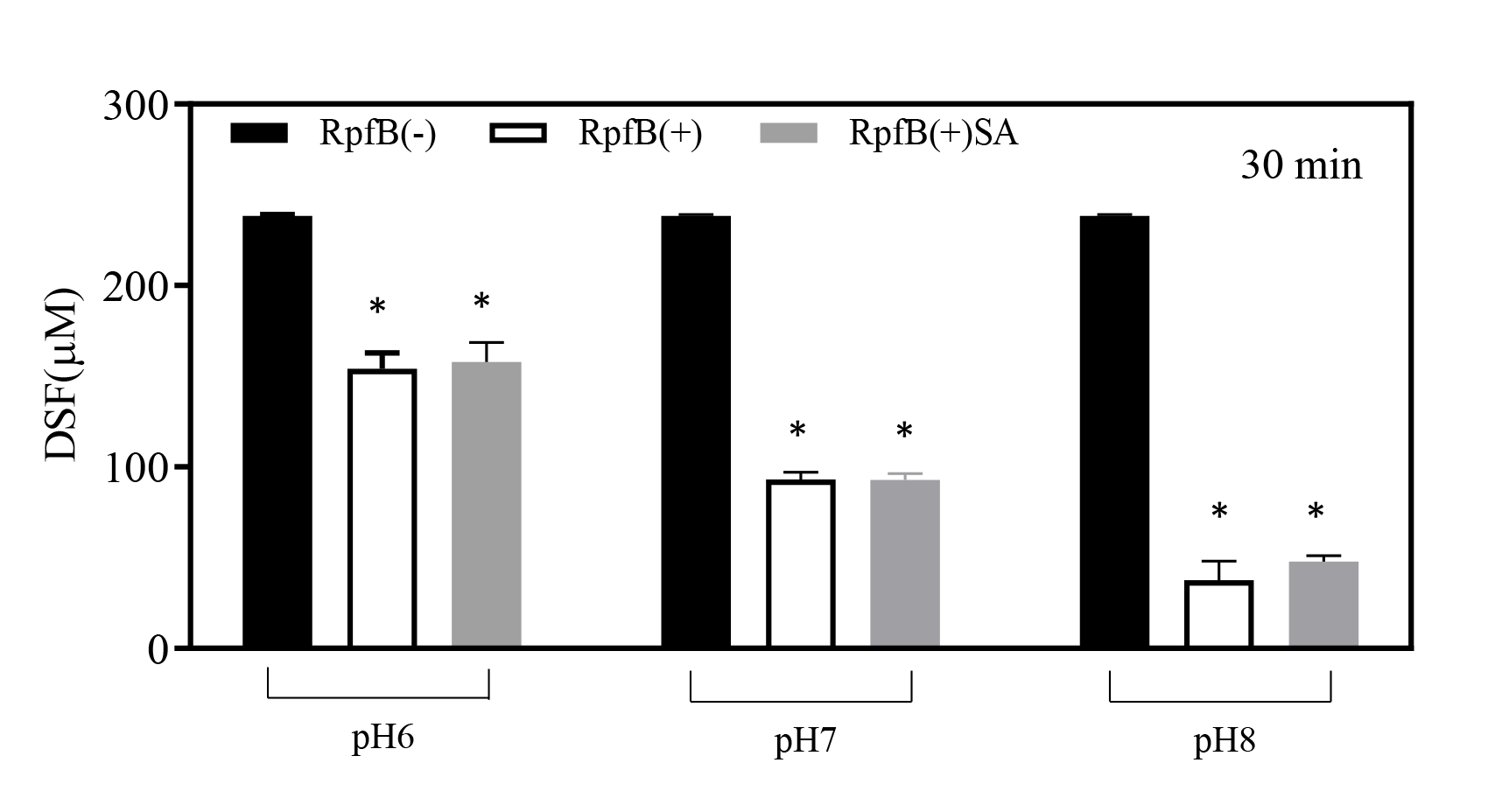

Supplement: FIG S6 [file mbio.03644-21-sf006.tif]

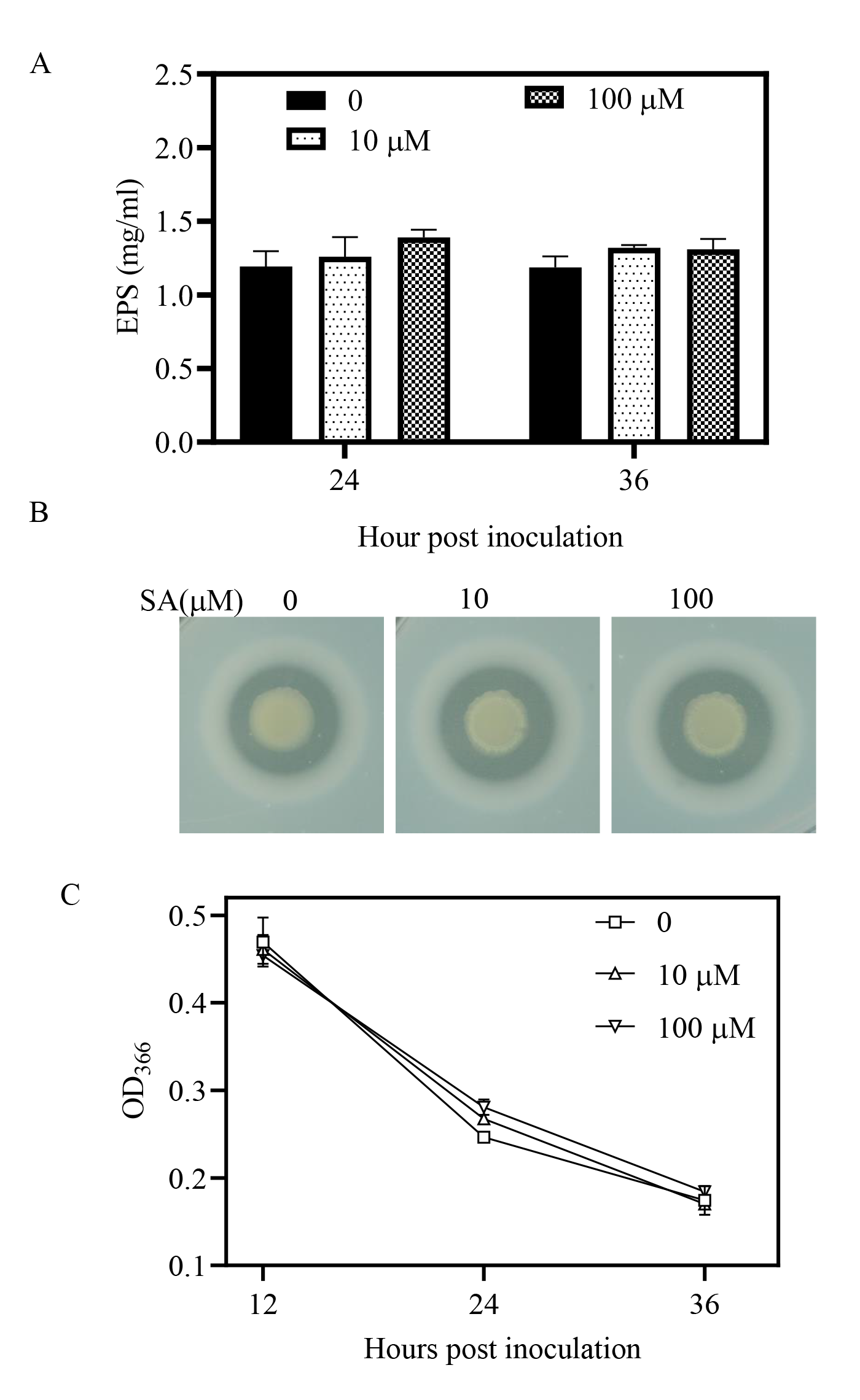

Supplement: FIG S7 [file mbio.03644-21-sf007.tif]
