# Supplementary material for: The Plant Defense Signal Salicylic Acid Activates the RpfB-Dependent Quorum Sensing Signal Turnover via Altering the Culture and Cytoplasmic pH in the Phytopathogen Xanthomonas campestris
Source: mBio. 2022 Mar 7;13(2):e03644-21. doi: 10.1128/mbio.03644-21 (PMC9040794; doi:10.1128/mbio.03644-21)
Supplement: TABLE S1 [file mbio.03644-21-st001.docx]

**Table S1. Bacterial strains, plasmids and oligos used in this study**

| **Strain** | **Properties/characteristics** | **Reference/source** |
| --- | --- | --- |
| ***Xcc* Strains** |  |  |
| XC1 | *Xcc* wild-type strain, Rif^R^ | Lab stock |
| Δ*rpfC* | The *rpfC* in-frame deletion mutant, Rif^R^ | He *et al*., 2006 |
| Δ*rpfB* | The *rpfB* in-frame deletion mutant, Rif^R^ | Zhou *et al*., 2015 |
| Δ*rpfB*Δ*rpfC* | The *rpfB rpfC* double deletion mutant, Rif^R^ | Zhou *et al*., 2015 |
| Δ*rpfB*::*rpfB* | The *rpfB* in-frame deletion mutant complemented with a single copy of *rpfB* inserted at the *att*Tn*7* site on its chromosome, Rif^R^ |  |
| Δ*rpfB*Δ*rpfC*::*rpfB* | The *rpfB rpfC* double deletion mutant complemented with a single copy of *rpfB* inserted at the *att*Tn*7* site on its chromosome, Rif^R^ | Zhou *et al*., 2015 |
| XC1::*pchAB* | The XC1 harboring a single copy of *pchAB* at the *att*Tn7 site on its chromosome, Rif^R^ | Cao *et al*., 2019 |
| Δ*rpfC*::*pchAB* | The Δ*rpfC* harboring a single copy of *pchAB* at the *att*Tn7 site on its chromosome, Rif^R^ | This study |
| XC1::P*_rpfB_*-*gusA* | The XC1 harboring a single copy of T0T1-P*_rpfB_*-*gusA* at the *att*Tn7 site on its chromosome, Rif^R^ | This study |
| XC1::P*_rpfF_*-*gusA* | The XC1 harboring a single copy of T0T1-P*_rpfF_*-*gusA* at the *att*Tn7 site on its chromosome, Rif^R^ | This study |
| XC1(pBBR-*mCherry-pHluorin*) | XC1 wide type harboring the expression plasmid pBBR-*mCherry-pHluorin*, Rif^R^ Kan^R^ | This study |
| Δ*rpfC*(pBBR-*mCherry-pHluorin*) | The *rpfC* in-frame deletion mutant harboring the expression plasmid pBBR-*mCherry-pHluorin*, Rif^R^ Kan^R^ | This study |
| ***E. coli* strains** |  |  |
| DH5α | *E. coli* F– Φ80lacZΔM15 Δ(lacZYA-argF) U169 recA1 endA1 hsdR17 (rK–, mK+) phoA supE44 λ– thi-1 gyrA96 relA1 | Lab stock |
| S17-1λpir | res^-^ pro mod^+^ integrated copy of RP4, mob^+^ | Lab stock |
| BL21(DE3) | *E. coli* B F- dcm ompT hsdS(r_B_- m_B_-) gal [malB^+^]_K-12_(λ^S^) | Lab stock |
| RK2013 | Triparental mating helper strain, Kan^R^ | Lab stock |

| **Plasmids** | **Properties / characteristics** | **Reference/source** | |
| --- | --- | --- | --- |
| pK18mobsacB | A mobilizable vector, allows for selection of double crossover in *Xcc*, Kan^R^ | | Schäfer *et al*., 1994 |
| pBBR1MCS-2 | Plasmid for gene complementation, Kan^R^ | | Kovach *et al*., 1995 |
| Pbbr1mcs-2-*pHluorin-mCherry* | *pHluorin-mCherry* cloned in pBBR1MCS-2, Kan^R^ | | This study |
| mini-Tn7T-Gm | a versatile mini-Tn7 delivery vector mini-Tn*7*T-Gm, Gm^R^ | | Choi and Schweizer, 2006 |
| mini-Tn7-T0T1-*gusA* | mini-Tn7T-Gm containing a T0T1 terminator fused to the coding region for *gusA*, Gm^R^ | | This study |
| mini-Tn7-T0T1-P*_rpfB_*-*gusA* | mini-Tn7T-Gm containing a T0T1 terminator and 512-bp promoter region of *rpfB* fused to the coding region for *gusA*, Gm^R^ | | This study |
| mini-Tn7-T0T1-P*_rpfF_*-*gusA* | mini-Tn7T-Gm containing a T0T1 terminator and 495-bp promoter region of *rpfF* fused to the coding region for *gusA*, Gm^R^ | | This study |
| pET-28a-*rpfB* | pET-28a containing *rpfB*, Kan^R^ | | Zhou *et al*., 2015 |

| **Application** | **Primers** | **Sequence (5’ to 3’)** |
| --- | --- | --- |
| RpfB protein expression | *rpfB*(pET)_F | GGAATTCcatatgATGAGTCAGGCACGTCCTTGGTTG |
|  | *rpfB*_R | CGggatccCTATGCCTTGGCCGCATCCC |
| pHluorin-mCherry protein expression by pBBR | pHluorin-F | CGgaattcATGGTTTCAAAAGGCGAAGAAGACAACATG |
|  | pHluorin-R | CGggatccTCATTTGTATAGTTCATCCATGCCATGTGTAATCC |

**Reference**

He Y W, Xu M, Lin K, et al. Genome scale analysis of diffusible signal factor regulon in *Xanthomonas campestris* pv. *campestris*: identification of novel cell–cell communication‐dependent genes and functions[J]. Molecular microbiology, 2006, 59(2): 610-622.

He Y W, Wang C, Zhou L, et al. Dual signaling functions of the hybrid sensor kinase RpfC of Xanthomonas campestris involve either phosphorelay or receiver domain-protein interaction[J]. Journal of Biological Chemistry, 2006, 281(44): 33414-33421.

Zhou L, Wang X Y, Sun S, et al. Identification and characterization of naturally occurring DSF‐family quorum sensing signal turnover system in the phytopathogen *Xanthomonas*[J]. Environmental microbiology, 2015, 17(11): 4646-4658.

Schäfer A, Tauch A, Jäger W, et al. Small mobilizable multi-purpose cloning vectors derived from the Escherichia coli plasmids pK18 and pK19: selection of defined deletions in the chromosome of Corynebacterium glutamicum[J]. Gene, 1994, 145(1): 69-73.

Kovach M E, Elzer P H, Hill D S, et al. Four new derivatives of the broad-host-range cloning vector pBBR1MCS, carrying different antibiotic-resistance cassettes[J]. Gene, 1995, 166(1): 175-176.

Choi K H, Schweizer H P. mini-Tn 7 insertion in bacteria with single *att*Tn7 sites: example Pseudomonas aeruginosa[J]. Nature protocols, 2006, 1(1): 153.
